# Meta-analyzing intelligence and religiosity associations: Evidence from the multiverse

**DOI:** 10.1371/journal.pone.0262699

**Published:** 2022-02-11

**Authors:** Florian Dürlinger, Jakob Pietschnig

**Affiliations:** Faculty of Psychology, Department of Developmental and Educational Psychology, University of Vienna, Vienna, Austria; Coventry University, UNITED KINGDOM

## Abstract

Over the past century, a remarkable body of research about the relationship of intelligence and religiosity has accumulated. So far, the majority of studies that investigated this relationship showed a negative correlation, indicating lower cognitive abilities of individuals reporting stronger religious beliefs. Although the effect direction has been observed to be largely consistent across studies, the reported effect strength varied substantially across studies. Several potentially moderating variables such as different intelligence and religiosity assessment methods, educational status of samples, and participant sex have been proposed as likely candidates for explaining systematic differences in effect strengths. However, the effects of these moderators are to date unclear. Consequently, we focused in investigating effects of these moderating variables on the intelligence and religiosity link in an update of prior meta-analytical investigations in *n* = 89 (*k* = 105; *N* = 201,457) studies. Random-effects analyses showed a small but robust negative association between intelligence and religiosity *r* = -.14 (*p* < .001; 95% CI [-.17, -.12]). Effects were stronger for (i) psychometric intelligence tests than for proxy measures such as grade point averages and (ii) general population and college samples than pre-college samples. Moreover, we provide evidence from combinatorial, multiverse, and specification curve analyses that further corroborates the robustness of the investigated association. Out of 192 reasonable specifications all 135 (70.4%) significant summary effects were negative. In all, our results show small but robust negative associations between religiosity and intelligence that are differentiated in strength but generalize in terms of direction over moderating variables.

## Introduction

Intelligence and religiosity associations have been investigated for more than 90 years by now. As early as in 1928, two studies [1,2] reported negative correlations between cognitive abilities and religiosity, indicating lower religiosity of more intelligent individuals. Subsequently, the association of intelligence and religiosity has been examined in several nations, by means of different intelligence measures, and with different methods to assess religiosity. On a national level, negative associations between intelligence and religiosity have been well-established.

Associations of national IQs with the respective countries’ atheism rates indicated higher test performance in countries with larger self-reported atheistic population percentages [3]. However, the investigation of potentially moderating variables within countries has so far led to less unequivocal results. For instance, these associations have been shown to be differentiated according to quality of life, yielding meaningful positive associations in countries with higher [4] but mostly nill-effects in countries with lower quality of life [5]. Moreover, within countries, the findings appear to be less consistent in terms of strength and in certain cases even of the sign, yielding seemingly erratic patterns of results possibly depending on subject-level variables such as participant sex, age, or highest educational qualification [6].

The first formal quantitative synthesis of the intelligence-religiosity association [6] found stronger negative effects in women than in men. A reanalysis of these very same data [5] suggested that the negative intelligence-religiosity link is limited to female samples only. However, in a more recent meta-analysis, a robust association that generalized over sex was identified [7]. Other inconsistencies between these meta-analytical accounts emerged as well, indicating either mediating effects of education [5] vs. no mediation [7] or partial [5] vs. full mediation [7] of intelligence on the education-religiosity link.

Effects of education on the intelligence and religiosity link are conceptually plausible because on the one hand, education is well-known to be robustly associated with intelligence [8] and on the other hand, highest educational attainment has been found to correlate negatively with formal church attendance [9].

Two potential causes may be assumed that make differences in the strength of the intelligence and religiosity association plausible. On the one hand, weaker associations in college samples than in general population samples may be attributed to range restriction. Samples comprising college students can be seen as more homogeneous than the general population in terms of cognitive ability, education, religiosity and other variables. Therefore, effects of college samples may be expected to be lower than those of less homogenous general population samples.

On the other hand, weaker associations of college and pre-college samples than general population samples may be seen as a function of participant ages. Religiosity has been shown to increase with age, although the increase appears to be non-linear because the most substantial changes take place in the early adulthood, but seems to further increase in old age [10]. However, so far, the majority of published studies examined intelligence and religiosity in samples with comparatively young participants (for some exceptions, see [11–13]). Nonetheless, the available meta-analyses [5–7] indeed reported weakest effects for pre-college samples, while (stronger) effects of college and non-college samples may not be as differentiated.

Besides such varying subject-level characteristics, primary studies examining the association of intelligence and religiosity also differ in their methods to assess both concepts. Many studies investigated correlations of psychometric intelligence test results with self-reported religiosity [e.g., 11]. However, in some studies, intelligence has only been measured by a proxy such as academic achievement (e.g., by means of Grade Point Averages; GPA) [e.g., 14], thus introducing more statistical noise in the cognitive ability assessment. These differences have been shown to impact the effect strength of the relation to religiosity [6], because GPA-based assessments represent more crude indicators of cognitive abilities than psychometric intelligence tests do. Therefore, effect sizes can be expected to systematically differ in regard to intelligence assessment types and consequently represent another meaningful moderating variable of the intelligence and religiosity link which needs to be taken into account when synthesizing effects.

Primary studies also differed in terms of their religiosity assessments. While some authors assessed religious beliefs directly by means of self-report questionnaires or single items [e.g. 15], others asked their probands about their engaging in certain religious behaviors such as participation in religious campus organizations [16], going to church [e.g. 17], or the amount of contact with and dependence on the church community [e.g. 18]. Assessing religious beliefs as compared to religious behavior has been shown to moderate the association with intelligence [6]. This seems plausible because religious behaviors are often motivated by non-religious reasons. The desire for social involvement, belonging to or acceptance in a community may lead to engaging in such behaviors and therefore constitute a less accurate estimate of religiosity [19]. Consequently, intelligence correlations with religious beliefs may expected to be stronger than those with religious behaviors.

Finally, meta-analytical effect estimations necessarily depend on several decisions that are made by the authors of a given meta-analyses, such as the definition of inclusion criteria, subgrouping, or the use of different analytical approaches. In regard to the present research question, this is illustrated by the differing results of prior meta-analyses that used identical data but arrived at different conclusions [5,6] because of their different conceptual and analytical choices. Consequently, there may be many different reasonable ways to synthesize a certain set of data that pertain to a given research question. It has been demonstrated that even seemingly arbitrary decisions in data treatment and analysis can have substantial influences on the outcomes [20,21]. Multiverse and specification curve analyses represent useful tools that allow the assessment of a large range of different reasonable meta-analytical summary effects [22].

## The present study

Here, we aim to investigate the strength of the intelligence and religiosity association by (i) assessing influences of specific conceptually plausible moderating and mediating variables and (ii) examining a large number of different reasonable approaches to synthesize this effect using multiverse and specification curve analyses. Moreover, we use several standard and novel methods to investigate potential dissemination bias in these data and examine evidence for cross-temporally declining effect sizes.

The study protocol including all hypotheses, sampling, and planned confirmatory analyses have been preregistered at https://osf.io/5r4qu prior to all data analyses (all deviations from the preregistered protocol are documented on the project page at https://osf.io/ka2ym/).

## Methods

### Literature search

**Fig 1:**
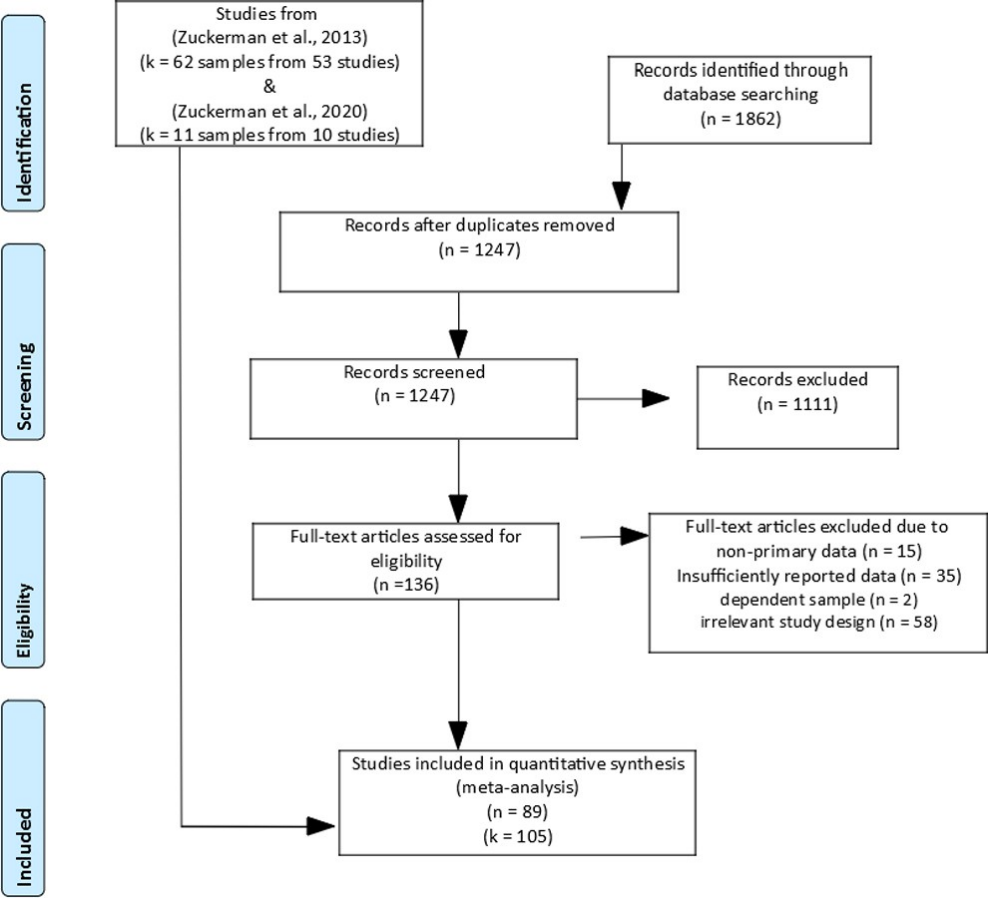
Flow-chart of study inclusion.

Potentially relevant articles were searched in five databases that index published journal articles and books (Google Scholar, ISI Web of Knowledge, PsycINFO, Pubmed, Scopus) as well as the Open Access Dissertation and Theses (oadt.org) which allows identification of relevant items from the grey literature. We used the following search string to identify titles and abstracts of potentially includable articles within the databases: (IQ OR intelligence OR “cognitive ability”) AND (religious OR religiosity OR “religious beliefs” OR spirituality). The title and abstracts of 1,862 potentially relevant articles were screened for relevance (for a flowchart, see Fig 1). Subsequently, full-texts of potentially relevant articles were obtained. The search process was originally carried out in July 2018 and was updated in September 2018. Another update was conducted in April 2021, applying the same search strategy.

### Inclusion criteria

Studies had to meet four criteria in order to be eligible for inclusion in the present meta-analysis. First, correlations between religiosity or spirituality with scores from psychometric intelligence tests or academic achievement measures had to be reported. Second, samples had to comprise healthy participants. Third, reported data had to be independent of data of other included studies. In cases of data dependencies, inclusion of (i) larger and (ii) more recently published samples was preferred. Finally, primary studies had to be published in either English or German. A list of included studies is available in the Online Supplement (S1 Appendix).

### Coding

Coding of studies into categories (type of religiosity measure: beliefs vs. behavior vs. mixed; type of intelligence measure: IQ vs. GPA vs. mixed; publication status: published vs. unpublished; sample type: pre-college vs. college vs. non-college) and recording of other relevant variables (publication year, effect sizes, their associated *p*-values) as well as sample characteristics (sample size, percentage of men within samples) was conducted twice by the same experienced researcher [FD]. Inconsistencies in coding were resolved by discussion with an independent researcher [JP]. Inclusion of correlations of religiosity with psychometric intelligence tests was preferred over those with less salient assessments of intelligence (e.g., GPA; if more than one correlation of a distinct measure was reported, correlations were averaged using *z*-transformations).

#### Primary study quality assessment

We assessed the quality of included studies by means of an adapted version of the Newcastle-Ottawa Quality Assessment Scale [23] for cross-sectional studies. Records were evaluated based on the representativeness of the sample, the sample size, handling of non-respondents as well as the extent of the response rate and ascertainment of the exposure, thus yielding an index of study quality ranging from 0 to 5 points. The adapted scale along with quality ratings for individual studies are detailed in the online supplementary materials (S2 Appendix).

### Data analysis

Effect sizes were synthesized by means of precision-weighted random-effects models. As a descriptive measure of heterogeneity, the index *I*^2^ was computed. This measure provides the percentage of the total variability which can be attributed to true variation between studies rather than sampling error. We interpreted *I*^2^-values ranging from 0 to 25% as indicative of trivial, 25–50% as small, 50–75% as moderate, and 75–100% as large heterogeneity in accordance with well-established guidelines [24].

Following common standards for synthesizing correlation coefficients, Pearson *r*s were transformed into Fisher`s *Z* prior to summary effect calculations. To facilitate interpretation, all effect sizes were back-transformed into the *r*-metric prior to reporting. All analyses were conducted using the open-source software R 3.1.1. and the packages metafor [25], robumeta [26], psych [27], pwr [28], puniform [29], plyr [30], ggplot2 [31], ggpubr [32], grid [33], and gridExtra [34].

#### Subgroup analyses

Potential influences of moderator variables were assessed using mixed-effects models (i.e., effect size estimates of each subgroup were based on random-effects models but between-subgroup comparisons were based on fixed-effect models). In a series of subgroup analyses, effect sizes were grouped according to the religiosity assessment type (beliefs vs. behavior vs. mixed), sample type (pre-college vs. college vs. non-college), and status of publication (published vs. unpublished). Moreover, we investigated potential influences of intelligence assessment type (categorized into: IQ vs. GPA vs. mixed assessment) in an exploratory (i.e., non-preregistered) analysis.

#### Meta-regression

Continuous moderators were examined by means of linear precision-weighted meta-regressions. Because recent evidence suggests overproportional numbers of declining effect strengths over time in empirical research regardless of the investigated research question [35], we examined potential influences of publication years on effect sizes. Therefore, we calculated two weighted meta-regressions for effect sizes on publication year based on all and on published effect sizes only. This approach was deemed appropriate because conceptually, decline effects are likely to be masked by results by the included unpublished effect sizes (see [35]). Moreover, effect sizes were regressed on percentage of men within samples for all studies for which sex breakups were available (*k* = 76). Finally, to test for a possible influence of primary study quality on reported estimates, meta-regressions of effect sizes on study quality were conducted.

#### Dissemination bias

We used several standard and more modern methods to assess potentially confounding effects of dissemination bias. Only published studies were included for dissemination bias analyses. First, funnel plots were visually inspected for indications of asymmetry. Second, Begg and Mazumdar`s rank correlational method [36] was used to formally examine possible funnel plot asymmetry. By means of this method, primary study effect sizes and their precision are ordinally correlated and interpreted as indicative of publication bias if associations become significant (here, an alpha level of .10 is assumed). Third, Sterne and Egger`s regression test [37] was employed. In this approach, standard normal deviates of effect sizes (the quotient of effect sizes and their standard errors) are regressed on study precision. In absence of publication bias, the intercept of the regression line should not significantly deviate from the origin.

Fourth, we used the Trim-and-Fill method [38] to estimate the number of missing studies on the asymmetric side of the funnel plot. Moreover, this approach provides an adjusted effect estimate which can be interpreted as a sensitivity analysis (i.e., large numerical deviations of standard and adjusted effect estimates indicate publication bias). Fifth, we tested for a potential excess of significant studies [39]. In this approach, the average power of the primary studies to detect the observed meta-analytic summary effect is calculated. Based on these power estimates, the expected frequencies of significant studies are calculated and compared with the number of observed significant studies (of note, signs of the significant primary study effect sizes need to be consistent with the summary effect) by means of a chi-squared test (i.e., significantly larger numbers of observed than expected studies being indicative of bias).

Finally, we used three novel methods for dissemination bias assessment (*p*-curve, *p*-uniform, *p*-uniform*) that are based on similar ideas, but rely on different implementations. These methods allow the estimation of meta-analytical summary effects that are based on the *p*-value distributions and their associated degrees of freedom of significant published primary studies only. To this end, it is argued that selective reporting of studies is driven by statistical significance rather than the strength of effect sizes (as for instance assumed in the Trim-and-Fill procedure) [40]. Because significant studies should not be vulnerable to strategic submission behaviors such as file-drawering (i.e., all significant studies have the same probability of being published), effect estimations that are only based on significant *p*-values can be expected to remain unaffected by dissemination bias.

Specifically, *p*-curve [41] relies on the observed distribution of independent significant *p*-values of a set of studies. Right skewed curves are indicative of evidential value of the observed set of studies (i.e., the meta-analytic summary effect differs significantly from zero), left skewed ones indicate *p*-hacking (i.e., a certain variety of questionable research practices; see [22]), and uniform distributions are indications of lacking evidential value (i.e., the accumulated evidence is insufficient to provide meaningful information about either the null or the alternative hypothesis). The *p*-curve distribution can be used to estimate summary effect sizes by recording the effect size that is associated with the theoretical density distribution of conditional *p*-values that fits best to the observed empirical *p*-value distribution.

Another method, which is based on the same underlying logic but differs in its implementation is *p*-uniform. In this approach, dissemination bias is assessed by examining potential left-skew of the conditional *p*-value distribution for a fixed-effect model [42]. For the summary effect estimation, the conditional *p*-values of the observed *p*-value distribution are uniformized and their associated effect estimate is assumed to represent the meta-analytical summary effect.

However, a drawback of both *p*-curve and *p*-uniform is their vulnerability for large between-studies heterogeneity, therefore limiting their applicability in many cases. Consequently, we additionally used *p*-uniform* [43] which is a recently developed extension of *p*-uniform that improves the effect estimation when considerable between-studies heterogeneity is observed.

In contrast to *p-*uniform, effect estimation in *p-*uniform* is based on the *p-*values of significant as well as non-significant studies and improves the previously established *p-*value-based estimation methods three ways, namely: (i) because of larger numbers of includable effect sizes, estimates become more accurate (i.e., they show smaller variance), (ii) overestimation of summary effects due to large between-studies heterogeneity is reduced, and (iii) the method allows a formal assessment of between-studies heterogeneity. Effect estimation in this method broadly resembles selection model approaches but does not require estimations of weight functions. Moreover, it is based on the assumption that significant studies on the one hand and non-significant studies on the other hand possess the same publication probability, although these probabilities may differ between these two study types [for details, see 44]. Of note, *p*-uniform does not provide a formal test for publication bias, but supposedly corrects for potential bias in its effect estimations. The R code for all our analyses is available in the Online Supplement (S3 Appendix).

#### Combinatorial meta-analysis

Another meta-analytic idea similar to the multiverse-analysis or the specification-curve approach is combinatorial meta-analysis [45]. This method aims at calculating effect estimates for all 2^k^ – 1 possible subsets of available data. It can be interpreted akin to sensitivity analyses, which are designed to identify outlier studies that overproportionally affect the summary effect estimates. On the one hand, this brute-force method is suited for identifying influential studies. On the other hand, it, blindly tests all possible study subsets, although most of them would not be considered as representing reasonable selections.

A maximum number of 105 available samples yields over 40 trillion unique subsets (2105–1 = 40,564,819,207,303,340,847,894,502,572,032) for an exhaustive combinatorial meta-analysis. We drew a random sample of 100,000 different subsets representative for the full set. To this end, we used a stratified approach to oversample studies with the smallest and largest effects to be able to meaningfully assess outlier influences.

Advantages of multiverse and specification curve analyses over combinatorial meta-analyses consist in their theoretical and conceptual proceeding as well as in their potential variation of the (meta-analytical) technique (e.g. fixed-effect vs. random-effects modelling).

#### Multiverse and specification curve analyses

In terms of the multiverse and specification curve analyses we distinguished between three internal, or “Which” factors (i.e., which data were meta-analyzed) and two external, or “How” factors (i.e., how were the data meta-analyzed).

The internal (i.e., “Which”) factors were: (i) type of religiosity assessment (beliefs, behavior, mixed, or a combination of all), (ii) sample type (college, non-college, pre-college, or a combination of all), and (iii) status of publication (published, unpublished, or a combination of both). Accordingly, these specifications yielded 4 * 4 * 3 = 48 combinations.

The external (i.e., “How”) factors were: (i) the choice of effect size (i.e., synthesis of Pearson *r*s vs. *Z*-transformed coefficients), (ii) estimator type (i.e., random-effects DerSimonian-Laird estimators vs. random-effects restricted maximum-likelihood estimators vs. fixed-effect estimators vs. unweighted estimation). These specifications yielded 3 * 4 = 12 different ways to analyze the same data.

In total, data and analysis specifications yielded 12 * 48 = 576 ways to include and analyze the data. We restricted analyses to unique combinations with at least two studies.

#### Inferential test of the specification-curve meta-analysis

To inferentially test if the (descriptive) meta-analytic specification curve indicated rejecting the null hypothesis of no effect, we used a parametric bootstrap approach. Study features for each primary study were regarded as fixed, but new effect sizes were assigned randomly assuming that the null hypothesis is true. In order to do so, values were drawn from a normal distribution with an expected value equivalent to zero, but a varying standard deviation, which corresponded to the sample’s observed standard error (obtained via a fixed-effect model). This specification-curve analysis was repeated 999 times. The resulting 1,000 bootstrapped specification curves were then used to obtain the pointwise lower and upper limits (2.5% and 97.5% respectively) for each specification number, which constitute the boundaries of the null hypothesis. When the limits did not include zero, the evidence was considered to be indicative of a non-nill summary effect.

#### Mediation analyses

A series of meta-analytical mediation models allowed us to investigate potential mediating effects of education and cognitive styles (i.e., analytic vs. intuitive) on the intelligence and religiosity link. We used random effects models and weighted least square estimations in the two-stage method for indirect effect estimation, following the approach of Cheung [46]. For these analyses, we used the R-package metaSEM [47].

### Final sample

Our final sample consisted of 89 studies comprising *k* = 105 independent samples (88 published vs. 17 unpublished effect sizes; *N* = 201,457) covering the time span from 1928 to 2020 (study characteristics are detailed in Table 1; the full data set is available at https://osf.io/ka2ym/). Most studies used psychometric intelligence tests to assess cognitive abilities (*k* = 93) whilst the rest used grade point averages as a proxies (*k* = 8) or a combination of both (*k* = 4). Most studies (*k* = 67) assessed self-reported religious beliefs, whilst the others used self-reported behaviors (*k* = 11) or a composite of beliefs and behaviors (*k* = 27). The majority of samples consisted of college students (*k* = 49), followed by general population (*k* = 39), pre-college samples (*k* = 13), and four mixed cases. A checklist of our study outline according to the PRISMA guidelines [48] can be accessed in the S4 Appendix.

**Table 1:** Details of included studies.

Author	Year	*N*	Effect size (*r*)	*p*-value	Sample type	Percentage of men within sample	Religiosity type	Intelligence measure	Publication status
Howells	1928	461	-.25	< .01	College	43	Beliefs	Thorndike Intelligence Test, Iowa Comprehension Test, and GPA	Published
Sinclair	1928	67	-.44	< .01	College	48	Beliefs	UEE	Published
Carlson	1934	100	-.19	.058	College	n/a	Beliefs	UEE	Published
Franzblau	1934	354	-.15	.005	Precollege	44	Beliefs	Terman Test of Mental Abilities	Published
(1)Symington	1935	200	-.24	.001	College	n/a	Beliefs	Otis Test of Mental Ability	Published
(2)Symington	1935	160	-.47	< .01	College	n/a	Beliefs	Otis Test of Mental Ability	Published
V.Jones	1938	268	-.24	< .01	College	n/a	Beliefs	UEE	Published
Corey	1940	234	-.03	.648	College	n/a	Beliefs	UEE	Published
Gilliland	1940	326	.00	1.000	College	n/a	Beliefs	Not specified	Published
Gragg	1942	100	-.02	.843	College	50	Beliefs	UEE	Published
Brown and Lowe	1951	108	-.43	< .01	College	n/a	Beliefs	UEE	Published
Dreger	1952	60	-.13	.322	Non-college	50	Beliefs	Wonderlic Personnel Test	Published
Kosa and Schommer	1961	361	.09	.088	College	100	Behavior	Assorted tests and GPA	Published
Hadden	1963	261	-.06	.334	College	n/a	Mixed	GPA	Published
Feather	1964	165	-.16	.040	College	100	Beliefs	Syllogisms	Published
Verhage	1964	1538	-.12	< .01	Non-college	n/a	Behavior	Groninger Intelligence Test	Published
(1)Young, Dustin and Holtzman	1966	481	.03	.512	College	69	Beliefs	GPA	Published
(2)Young, Dustin and Holtzman	1966	574	-.11	.008	College	57	Beliefs	GPA	Published
Feather	1967	40	-.09	.581	College	50	Behavior	Syllogisms	Published
Bender	1968	96	-.10	.332	Non-college	100	Behavior	UEE and GPA	Published
Southern and Plant	1968	72	-.75	< .01	Non-college	58	Beliefs	Mensa membership	Published
(1)Hoge	1969	179	-.12	.110	College	n/a	Mixed	UEE	Unpublished
(2)Hoge	1969	135	-.08	.356	College	n/a	Mixed	UEE	Unpublished
(3)Hoge	1969	327	-.07	.207	College	n/a	Mixed	UEE	Unpublished
Kahoe	1974	188	.18	.001	College	38	Beliefs	GPA / American College	Published
(1)Salter and Routledge	1974	339	-.15	.006	College	n/a	Beliefs	UEE	Published
(2)Salter and Routledge	1974	241	-.18	.005	College	n/a	Beliefs	UEE	Published
Foy	1975	36	-.50	.002	Non-college	50	Beliefs	WAIS	Unpublished
Poythress	1975	195	-.19	.008	College	n/a	Beliefs	UEE	Published
Dodrill	1976	44	.05	.747	Non-college	54	Beliefs	WAIS	Published
Francis	1979	2272	.04	.057	Precollege	n/a	Mixed	IQ from school records	Published
(1)Turner	1980	200	-.04	.574	Precollege	100	Beliefs	Thurstone Primary Mental Abilities Scale	Published
(2)Turner	1980	200	-.02	.779	Precollege	100	Beliefs	Thurstone Primary Mental Abilities Scale	Published
Francis, Pearson and Stubbs	1985	290	-.13	.055	Precollege	72	Beliefs	IQ (not specified)	Published
Francis;Francis	1997	711	-.04	.287	Precollege	40	Mixed	Raven Progressive Matrices	Published
(1)Blanchard-Fields, Hertzog, Stein and Pak	2001	96	.04	1.000	College	60	Mixed	Shipley Vocabulary Test	Published
(2)Blanchard-Fields, Hertzog, Stein and Pak	2001	219	-.32	< .01	Non-college	42	Mixed	Shipley Vocabulary Test	Published
Crossman	2001	75	-.36	.002	Non-college	0	Beliefs	Immediate free recall	Unpublished
Saroglou and Scariot	2002	94	.13	.212	Precollege	41	Mixed	GPA	Published
Horowitz and Garber	2003	172	.05	.515	Precollege	46	Mixed	WISC Vocabulary and Block design	Published
Saroglou and Fiasse	2003	120	.07	.447	College	56	Beliefs	GPA	Published
Clark	2004	77	-.12	.299	College	22	Beliefs	WAIS III	Published
Carothers, Borkowski, Burke, Lefever and Whitman	2005	101	-.25	.012	Non-college	0	Behavior	WAIS-R Vocabulary and Block design	Published
Ciesielski-Kaiser	2005	216	-.14	.040	College	36	Beliefs	Shipley Institute for Living Scale	Unpublished
Wahling	2005	35	-.32	.061	Non-college	14	Beliefs	Six cognitive ability tests	Unpublished
Hergovich and Arendasy	2005	180	-.23	.002	College	41	Beliefs	Wiener Matrizen-Test	Published
McCullough, Enders, Brion and Jain	2005	951	-.45	< .01	Non-college	n/a	Beliefs	Stanford-Binet	Published
Deptula, Henry, Shoeny and Slavick	2006	11963	-.10	< .01	Precollege	n/a	Beliefs	Modified Peabody Picture Vocabulary Test	Published
Räsänen, Tirri and Nokelainen	2006	142	-.17	.043	Precollege	n/a	Beliefs	Assorted tests	Published
Cottone, Drucker and Javier	2007	123	-.14	.122	College	35	Beliefs	WAIS III comprehension and similarities and GPA	Published
(1)Stanovich and West	2007	439	-.24	< .01	College	24	Beliefs	UEE	Published
(2)Stanovich and West	2007	1045	-.18	< .01	College	31	Beliefs	UEE	Published
Szobot et al.	2007	236	.15	.021	Precollege	100	Behavior	WAIS III block design and Vocabulary	Published
Bloodgood, Turnley and Mudrack	2008	230	-.15	.023	College	63	Behavior	UEE	Published
Bertsch and Pesta	2009	278	-.15	.012	College	42	Beliefs	Wonderlic Personnel Test	Published
Inzlicht, McGregor, Hirsh and Nash	2009	22	-.13	.564	College	41	Beliefs	Wonderlic Personnel Test	Published
Nyborg	2009	3742	-.05	.002	Precollege	n/a	Behavior	Assorted tests	Published
(1) Boazman	2010	122	.13	.154	College	52	Mixed	GPA	Unpublished
(2) Boazman	2010	91	-.26	.013	College	34	Mixed	GPA	Unpublished
(1)Kanazawa	2010	14277	-.12	< .01	Non-college	47	Beliefs	Peabody Picture Vocabulary Test	Published
(2)Kanazawa	2010	7160	-.14	< .01	Non-college	44	Beliefs	Verbal synonyms	Published
Nokelainen and Tirri	2010	20	-.20	.398	Precollege	45	Beliefs	WAIS III	Published
Raman	2010	129	.15	.090	College	n/a	Mixed	ACER Word Knowledge test	Unpublished
Sherkat	2010	12994	-.15	< .01	Non-college	43	Mixed	Vocabulary Test	Published
Lewis, Ritchie and Bates	2011	2155	-.16	< .01	Non-college	n/a	Mixed	Assorted tests	Published
Heaven, Ciarrochi and Leeson	2011	375	-.14	.007	Precollege	45	Beliefs	G of six numerical and three verbal tests	Published
Shenav, Rand and Greene	2011	306	-.06	.295	College	35	Beliefs	Shipley Vocabulary Test, and WAIS III Matrix Reasoning Test	Published
Sherkat	2011	1780	-.34	< .01	Non-college	n/a	Beliefs	Scientific Literacy Scale from General Social Survey	Published
(1)Pennycook, Cheyne, Seli, Koehler and Fugelsang	2012	223	-.19	.004	Non-college	41	Mixed	Assorted tests	Published
(2)Pennycook, Cheyne, Seli, Koehler and Fugelsang	2012	267	-.17	.005	Non-college	22	Mixed	Assorted tests	Published
Ganzach and Gotlibovski	2013	8984	-.23	< .01	Mixeda	n/a	Beliefs	AFQT (Armed Forces Qualifying Test)	Published
Pennycook and Cheyne, Koehler and Fugelsang	2013	91	-.34	.001	College	27	Beliefs	WordSum	Published
Razmyar and Reeve	2013	150	-.16	.050	College	47	Beliefs	Employee Aptitude Survey—abbreviated	Published
Ritchie, Gow and Deary	2014	550	-.15	< .01	Non-college	43	Beliefs	Raven`s Standard Progressive Matrizes, phonemic verbal fluency, logical memory from the Wechsler memory Scale- Revised	Published
Pennycook, Cheyne, Barr, Koehler and Fugelsang	2014a	505	-.27	< .01	Non-college	52	Beliefs	Numeracy, WordSum	Published
Pennycook, Cheyne, Barr, Koehler and Fugelsang	2014b	198	-.23	.001	College	32	Beliefs	WordSum	Published
Sacher	2015	44	-.01	.949	College	27	Beliefs	Shipley-2 abbreviated test of intelligence	Unpublished
Ross	2015	558	-.14	.001	Non-college	48	Mixed	Numeracy, WordSum, Syllogisms	Unpublished
Kirkegaard and Bjerrekaer	2016	37078	-.26	< .001	Non-college	66	Beliefs	Latent factor of several items	Published
Pennycook, Ross, Koehler and Fugelsang	2016	1065	-.16	< .001	College	29	Beliefs	Numeracy, WordSum	Published
Zuckerman and McPhetres	2016	1477	-.25	< .001	Non-college	27	Beliefs	Verbal, numerical and spatial tests	Unpublished
Saribay and Yilmaz	2017	426	-.10	.039	Non-college	38	Beliefs	WordSum, Base rate neutral problems	Published
(1) Daws and Hampshire	2017	30762	-.10	< .001	Non-college	n/a	Behavior	Overall score for 12 tests	Published
(2) Daws and Hampshire	2017	15843	-.14	< .001	Non-college	n/a	Behavior	Overall score for 13 tests	Published
Hartman, Dieckmann, Sprenger, Stastny and DeMarree	2017	598	-.20	< .001	Non-college	35	Beliefs	Numeracy, Shipley 2 tests	Published
Pollet and Schnell	2017	475	-.22	< .001	Non-college	57	Beliefs	Fluid intelligence	Published
Stankov and Lee	2018	8883	-.19	< .01	College	41	Mixed	Number series test	Published
(1) Strimaitis	2018	110	-.04	.686	College	31	Beliefs	Numeracy	Unpublished
(2) Strimaitis	2018	185	-.14	.057	College	20	Beliefs	Numeracy	Unpublished
(1) Drewelies, Deeg, Huisman and Gerstorf	2018	795	.00	1.000	Non-college	n/a	Beliefs	15-Words Test	Published
(2) Drewelies, Deeg, Huisman and Gerstorf	2018	819	-.06	.086	Non-college	n/a	Beliefs	15-Words Test	Published
Erlandsson, Nilsson, Tinghög and Västfjäll	2018	1015	-.23	.001	Non-college	50	Behavior	Numeracy	Published
Leonard	2018	266	-.16	.008	Mixed	45	Beliefs	Raven`s Advanced Progressive Matrices	Unpublished
Foong, Hamid, Ibrahim and Haron	2018	2322	.07	.002	Non-college	48	Beliefs	Montreal Cognitive Assessment	Published
Ståhl and van Prooijen	2018	322	-.17	.002	Non-college	53	Mixed	Numeracy, WordSum	Published
Perales	2018	11654	-.08	< .001	Non-college	46	Beliefs	Verbal, matching symbols, and memory test	Unpublished
Cavojová, Šrol and Jurkovič	2019	317	-.26	.001	Mixed	41	Beliefs	Wiener Matrizen-Test	Published
Cavojová, Secară, Jurkovič and Šrol	2019	121	.19	.194	Mixed	23	Mixed	Wiener Matrizen-Test	Published
(1) Lowicki, Zajenkowski and van der Linden	2019	301	-.17	.004	Non-college	36	Beliefs	Catell`s Culture Fair Intelligence Test 3	Published
(2) Lowicki, Zajenkowski and van der Linden	2019	200	-.07	.326	Non-college	53	Beliefs	Catell`s Culture Fair Intelligence Test 3, Number Series Test, Paper Folding Test	Published
Nilsson, Erlandsson and Västfjäll	2019	985	-.16	.001	Non-college	50	Mixed	Numeracy	Published
(1) Patel, Baker and Scherer	2019	539	-.08	.064	College	n/a	Beliefs	Rasch Numeracy Scale	Published
(2) Patel, Baker and Scherer	2019	631	-.13	.002	College	38	Beliefs	Rasch Numeracy Scale	Published
Betsch, Aßmann and Glöckner	2020	599	-.22	< .01	Non-college	40	Beliefs	Numeracy	Published
Furnham and Grover	2020	475	-.11	.020	Non-college	51	Beliefs	n/a	Published

*Note*. UEE = University Entrance Exams; GPA = Grade Point Average; n/a = no available; WAIS = Wechsler Adult Intelligence Scale; WISC = Wechsler Intelligence Scale for Children; ACER = Australian Council for Educational research.

aParticipants were 15 years old on average when intelligence was measured and 26 years old when religiosity was assessed.

## Results

**Fig 2:**
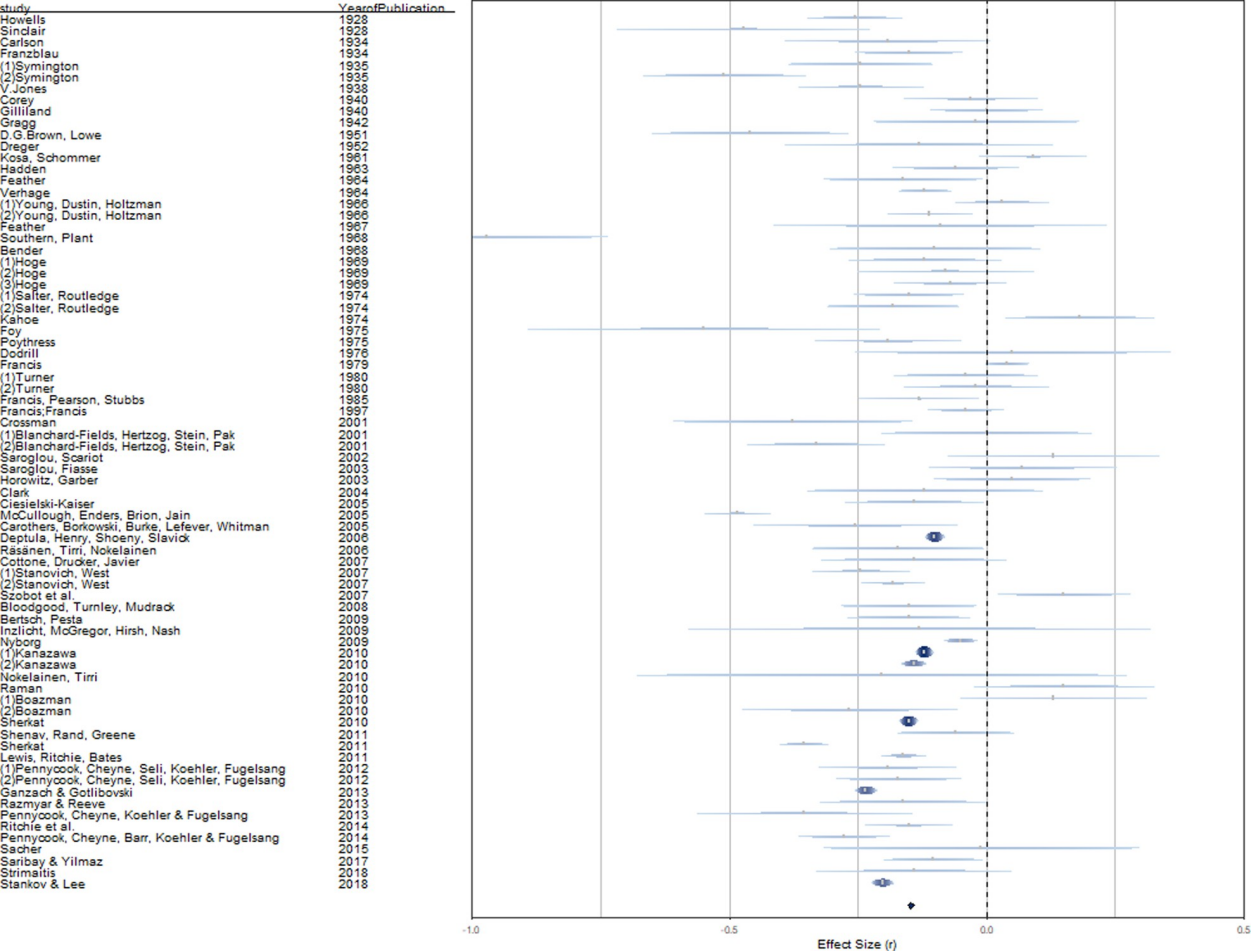
Rainforest plot for associations of intelligence measures with religiosity. *Note*. Overall effect size calculations are based on random-effects models; the diamond represents the summary effect size; length of confidence intervals varies according to relative study weights within the analysis.

Random-effects analyses yielded an overall effect of *r* = -.14 (*p* < .001; 95% CI [-.17, -.12]; see first line in Table 2). We provide a rainforest plot of our main analysis in Fig 2 [for details, see 49]. Between-studies heterogeneity was substantial (*Q* = 1462.27, *p* < .001, *I*^2^ = 96.12%, *τ*^2^ = 0.018, SE = 0.013), suggesting the presence of unobserved heterogeneity that may be explained by moderating variables. Outlier analyses by means of influence diagnostics (standardized residuals, DFFITS values, Cook`s distances, covariate ratios, leave-one-out values for heterogeneity test statistics, hat values, weights; [50]) revealed two leverage points (S5 Appendix). However, here we report results based on all available data points because recalculating our models without the leverage points yielded virtually identical results. Considering specification curve and multiverse analysis, this constitutes mere two specifications, while there are various alternative specifications.

**Table 2:** Random-effects estimates of overall data and according to intelligence assessment type, religiosity assessment, sample type, and publication status.

	Summary effect (*r*)	*SD*	*p*-values	95% *CI*	*Q*	*I* ^2^
Overall (k = 105)	-.141	0.013	< .001	[-.167, -.116]	1462.271***	96.12%
Intelligence assessment						
IQ (k = 93)	-.154	0.013	< .001	[-.180, -.130]	1371.209***	95.95%
GPA (k = 8)	-.011	0.048	.819	[-.084, .106]	24.674**	73.91%
Mixed assessment (k = 4)	-.100	0.081	.213	[-.254, .058]	24.158***	82.39%
Religiosity assessment						
All assessments						
Beliefs (k = 67)	-.167	0.018	< .001	[-.199, -.131]	1031.528***	95.94%
Behavior (k = 11)	-.090	0.036	.013	[-.160, .019]	79.968***	97.17%
Mixed (k = 27)	-.109	0.021	< .001	[-.150, -.067]	181.439***	89.78%
IQ-tests only						
Beliefs (k = 61)	-.177	0.018	< .001	[-.211, -.143]	974.030***	95.76%
Behavior (k = 9)	-.109	0.036	.002	[-.179, -.039]	65.506***	97.16%
Mixed (k = 23)	-.121	0.021	< .001	[-.161, -.080]	162.289***	89.34%
Sample type						
All assessments						
Pre-college (k = 13)	-.038	0.027	.165	[-.091, .016]	39.738***	75.59%
College (k = 49)	-.133	0.019	< .001	[-.170, -.095]	222.728***	87.33%
Non-college (k = 39)	-.177	0.022	< .001	[-.218, -.135]	989.095***	98.13%
IQ-tests only						
Precollege (k = 12)	-.046	0.028	.096	[-.099, .008]	37.402***	75.82%
College (k = 39)	-.158	0.018	< .001	[-.193, -.123]	145.650***	81.41%
Non-college (k = 38)	-.179	0.022	< .001	[-.221, -.136]	988.774***	98.22%
Publications status						
All assessments						
Published (k = 88)	-.145	0.015	< .001	[-.172, -.116]	1342.887***	96.57%
Unpublished (k = 17)	-.127	0.032	< .001	[-.188, -.065]	74.455***	81.28%
IQ-tests only						
Published (k = 78)	-.158	0.015	< .001	[-.186, -.130]	1259.927***	96.48%
Unpublished (k = 15)	-.134	0.030	< .001	[-.191, -.076]	65.627**	76.55%

*Note*. *SD* = standard deviation; 95% *CI* = 95% lower and upper bound of 95% confidence interval; *Q* = Cochran`s *Q* test statistic for heterogeneity; *I*^2^ = ratio between true heterogeneity and total observed variation

**p < .01

***p < .001.

### Combinatorial meta-analysis

**Fig 3:**
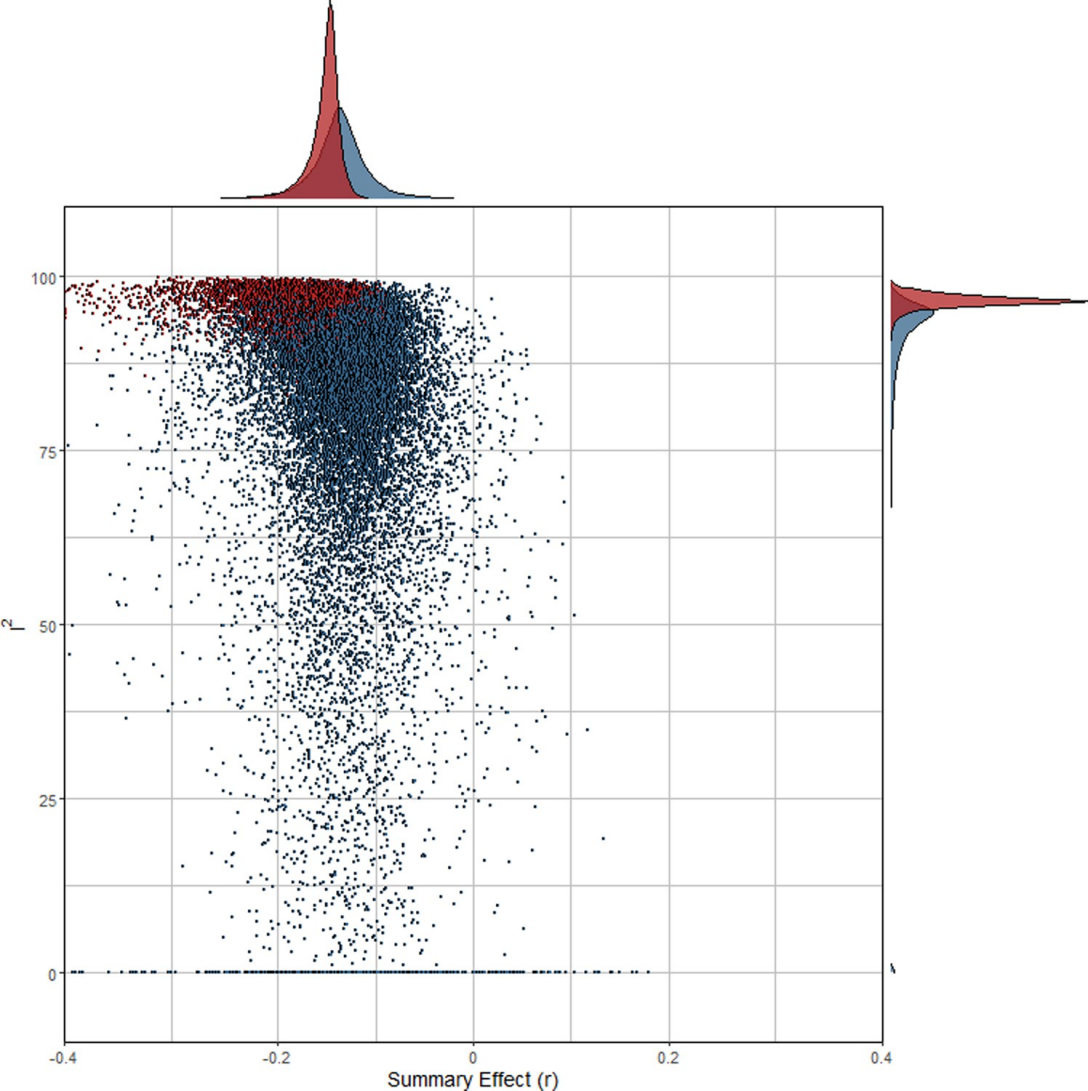
GOSH plot for combinatorial meta-analysis. *Note*. Each dot represents the summary effect of a random subset of studies. A random sample of 100,000 different subsets is depicted; subset estimations including at least one of the leverage points are highlighted in red.

In Fig 3 the combinatorial meta-analyses are visualized (GOSH plot). As mentioned earlier, we drew 100,000 random subsets from a larger number of theoretically possible subsets. In general, effect heterogeneity was high, especially when at least one of the two outliers was included (highlighted in red). Clearly, the outlying studies contributed considerably to overall-heterogeneity which was supported by density estimates of the effect distributions. The density estimates of the effect size considerably deviated from zero, suggesting a true non-nill effect.

### Multiverse and specification-curve analysis

**Fig 4:**
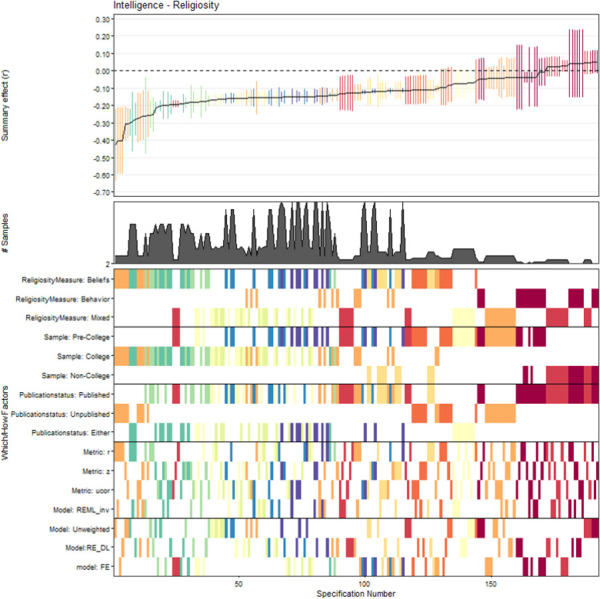
Descriptive meta-analytic specification-curve plot. *Note*. Specifications`summary effects with their associated 95% confidence intervals are illustrated sorted by magnitude. Directly below is the number of samples contained in the corresponding meta-analytic specification displayed, and below that one can see the combination of Which and How factors constituting each specification. Colors in this pattern indicate the number of samples included in the corresponding specification. Hot colors (red, orange, yellow) indicate that very few samples constitute the respective specification, whereas cool colors (blue, green, violet) indicate a larger number of samples in a given specification. The combinations of Which and How factors constituting each specification are displayed in the lower part. Corresponding summary effects are shown in the upper part.

**Fig 5:**
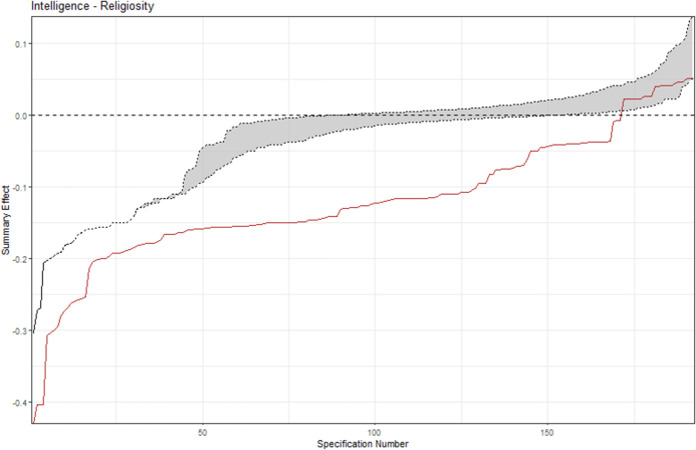
Inferential meta-analytic specification plot. *Note*. The specification curve (red) of the effect strength-sorted observed meta-analytic summary effects for all specifications is compared to the under-the-null scenario of a possible zero effect (grey).

**Fig 6:**
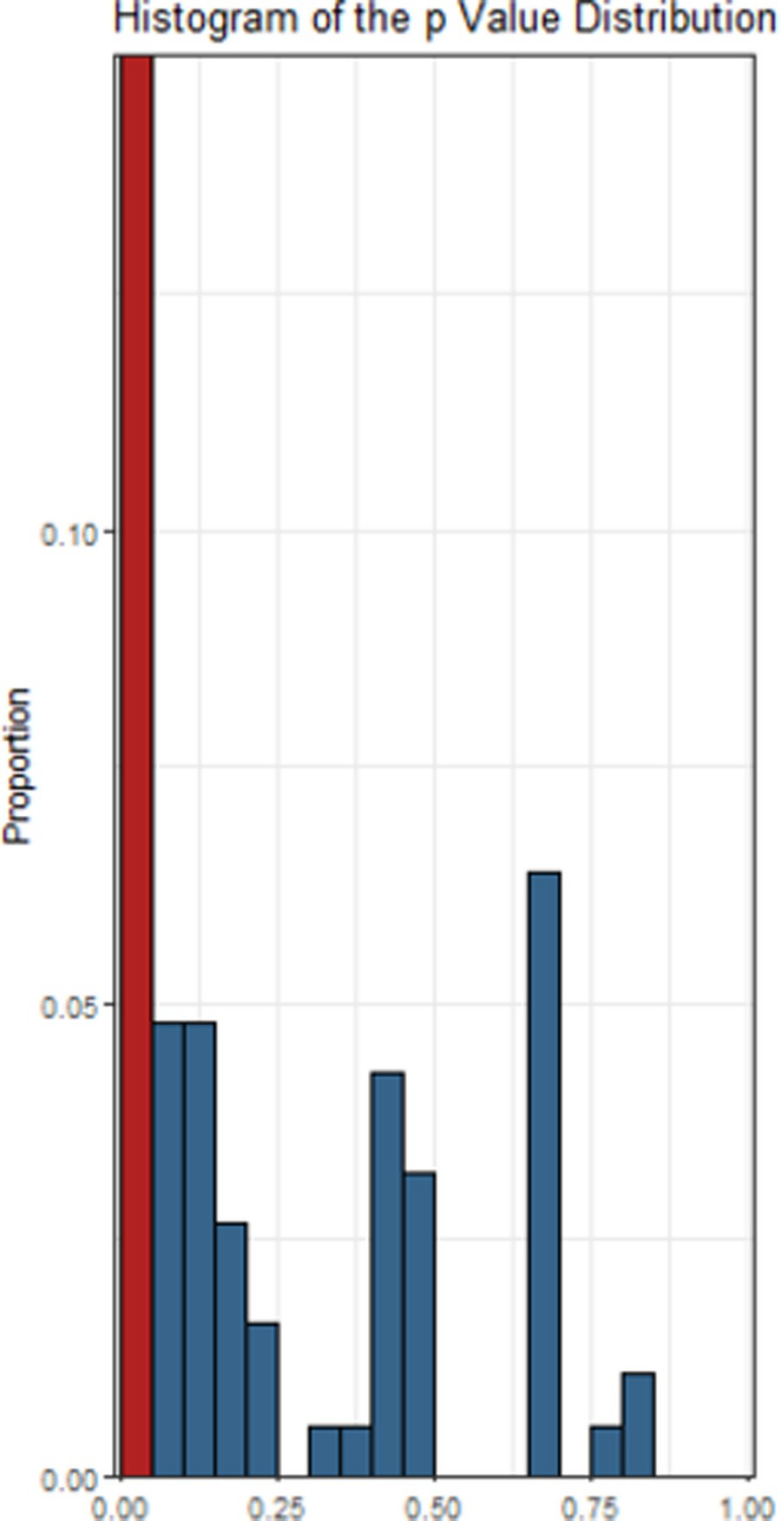
Histograms of p values for all meta-analytic specifications. *Note*. The proportion of nominally significant values (*p* < .05) is highlighted in red.

The descriptive meta-analytic specification-curve plot is provided in Fig 4. Almost all meta-analytic specifications yielded a negative summary effect. Out of 576 possible specifications, 192 comprised more than a single study, yielding 135 (70.4%) nominally significant (*p* < .05) negative summary effects. None of these specifications led to a significant positive effect. The observed results clearly deviate from the under-the-null scenario of an underlying zero effect (Fig 5), indicating a robust negative association. Fig 6 displays the histogram of the *p*-value distribution for the summary effect of the various meta-analytic specifications. There is an obvious excess of *p*-values smaller than .05, further corroborating the above evidence.

### Moderator analyses

Descriptive statistics of summary effects of all subgroup analyses are provided in Table 2.

#### Intelligence assessments

Mixed-effects models were used to investigate if religiosity associations with standardized intelligence test scores differed from those with academic achievement (as assessed by Grade Point Average: GPA). Religiosity showed significantly stronger associations with intelligence test scores than with GPA-based assessments (*Q* = 11.03, *df* = 1; *p* < .001). Associations with mixed assessments yielded correlation strengths in-between psychometric intelligence test score and GPA-only assessments although neither difference reached nominal statistical significance (IQ vs. mixed: *Q* = 0.45, *df* = 1; *p* = .50; GPA vs. mixed: *Q* = 1.41, *df* = 1; *p* = .24).

Consequently, we report all further analyses for both overall data and the subset of studies that used psychometric intelligence assessments-only to account for potential differential effects of moderating influences of assessment type (because of small case numbers, no separate analyses are provided for GPA-only or mixed assessments).

#### Religiosity assessments

Intelligence and religiosity associations differed significantly according to religiosity assessment type (*Q* = 4.62, *df* = 1, *p* = .032). Pairwise comparisons of studies assessing beliefs and studies assessing religious behaviors yielded marginally significant differences (*Q* = 3.64, *df* = 1; *p* = .056). Associations with mixed religiosity assessments yielded significantly lower correlations than those with beliefs (*Q* = 4.27, *df* = 1; *p* = .04) but no differences compared to those with behaviors (*Q* < 0.01, *df* = 1; *p* = .64). Follow-up analyses of the psychometric intelligence test subset yielded virtually identical results (beliefs vs. behavior: *Q* = 2.94, *df* = 1; *p* = .09; beliefs vs. mixed: *Q* = 4.29, *df* = 1; *p* = .04; behavior vs. mixed: *Q* = 0.08, *df* = 1; *p* = .78).

#### Sample type and sex

Associations appeared to be differentiated according to the educational status of the assessed samples (*Q* = 15.34, *df* = 1, *p* < .001). General population correlations were strongest, yielding absolute larger effects than pre-college (*Q* = 16.11, *df* = 1; *p* < .001) and (although not nominally significant) college samples (*Q* = 2.41, *df* = 1; *p* = .12). Moreover, college sample associations were larger than those of pre-college samples (*Q* = 8.08, *df* = 1; *p* < .01). Results of the subset of primary studies that used psychometric intelligence tests yielded consistent results in our subgroup analyses (precollege vs. non-college: *Q* = 14.46, *df* = 1; *p* < .001; college vs. non-college: *Q* = 0.54, *df* = 1; *p* = .46; precollege vs. college: *Q* = 11.93, *df* = 1; *p* = < .001).

We corrected our effect estimations for range restriction of intelligence in college samples using Thorndike`s Case 2 formula [51] following the approach of Zuckerman and colleagues [7] (we assumed a ratio of 1/.67 for students that applied but did not attend vs. students that applied and did attend college). Consequently, intelligence and religiosity correlations were corrected for range restriction before estimating the overall effect for college samples. Effects were once more differentiated (*Q* = 15.34, *df* = 1, *p* < .001), yielding *r* = -.20; *p* < .001; 95% *CI* [-.26; -.14]. The absolute effect was significantly larger than the pre-college estimate (*r* = -.04; *Q* = 16.02, *df* = 1, *p* < .001), but did not differ from the general population summary effect (*r* = -.18; *Q* = 0.44, *df* = 1, *p* = .51).

**Fig 7:**
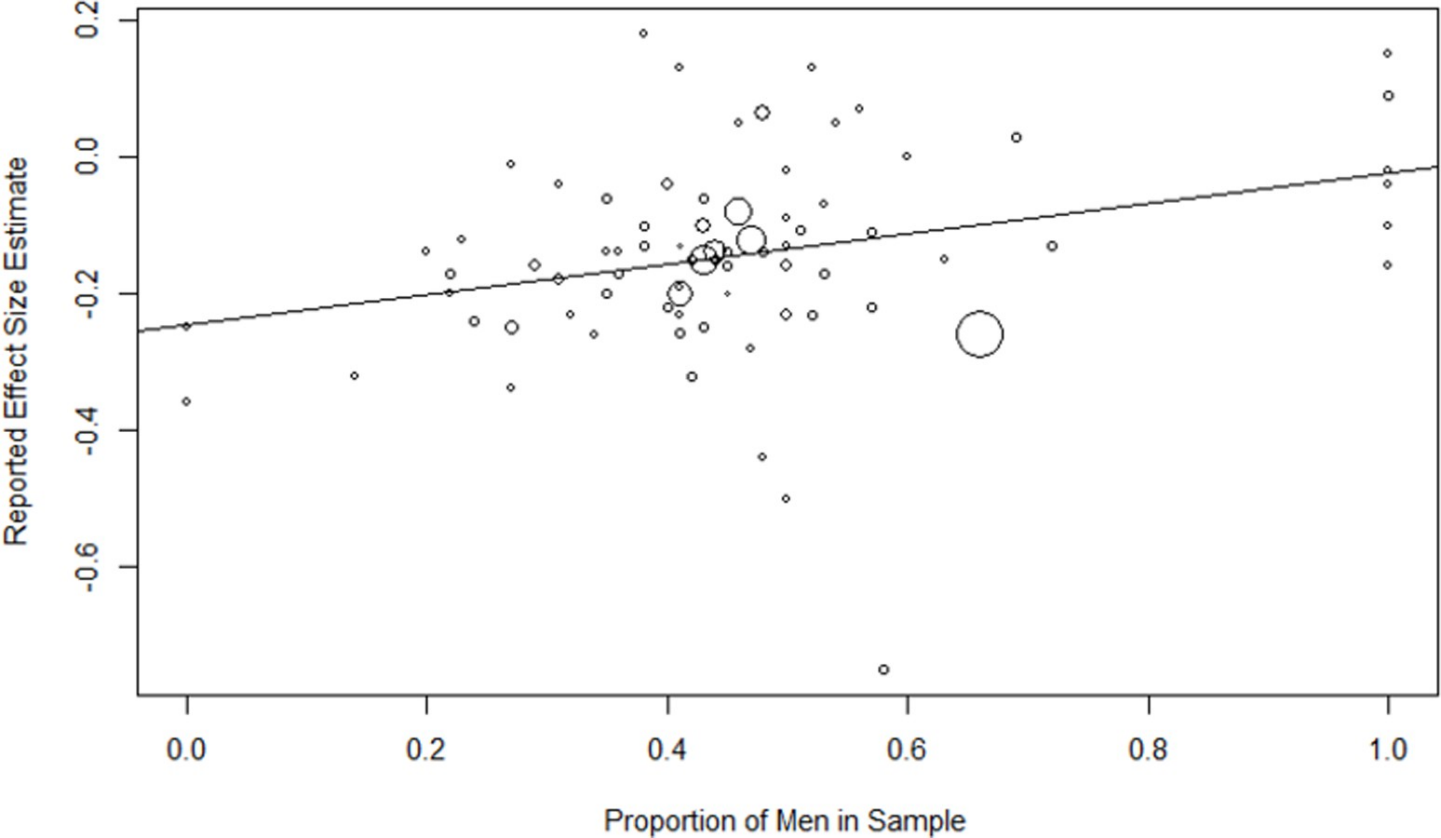
Meta-regression of percentage of men in samples on effect size*s*. Symbol size*s are* varied according to the relative study weights.

A meta-regression of effect sizes on the proportion of men (Fig 7) revealed a significant positive influence on sex (*b* = 0.22; *R*^2^ = .18, *p* = .002), indicating stronger links of intelligence and religiosity in men than in women. When conducting this meta-regression solely with studies using psychometric intelligence tests, the influence remained significant (*b* = 0.18; *R*^2^ = .12, *p* = .015).

Subgroup analyses between the six samples consisting solely of men (*r =* -.01; *p =* .97; 95% CI [-.10, .09]) and of the two comprising exclusively women (*r =* -.30; *p <* .001; 95% CI [-.43, -.16]) contrasted our results from regression analyses. Mixed-effects subgroup analyses revealed significant differences (*Q* = 11.32, *df* = 1; *p* < .001), indicating larger effects for women than for men.

#### Publication type

Differences in effect size estimates of published and unpublished studies conformed to the expected direction, showing smaller (albeit nominally non-significant) effects for unpublished studies (*Q* = 0.24, *df* = 1; *p* = .62). Again, results of studies that only used psychometric intelligence test scores were virtually identical, showing non-significantly larger absolute effects of published than unpublished studies (*Q* = 0.54, *df* = 1; *p* = .46). However, non-significant results of direct comparisons between published and unpublished effect sizes were to be expected, because of the (typically) considerably smaller number of unpublished studies (83.81% published vs. 16.19% unpublished) and the corresponding large confidence interval of their summary effect.

#### Multiple moderation analysis

We tested the effects of sex, year, and publication status in a multiple moderation analysis. No significant influences of these variables on the intelligence and religiosity link were observed (proportion of men: *b* = -0.217 [-.847; .414], *p* = .50, year of publication: *b* = -0.002 [-.005; .001], *p* = .18, status of publication: *b* = -0.071 [-.254; .112], *p* = .45). Moreover, none of these variables had an effect in simple moderation analyses (except in certain cases for sex, which showed seemingly erratic patterns of moderating influences), thus further corroborating the remarkable generalizability of the intelligence and religiosity link across moderators.

#### Dissemination bias

**Fig 8:**
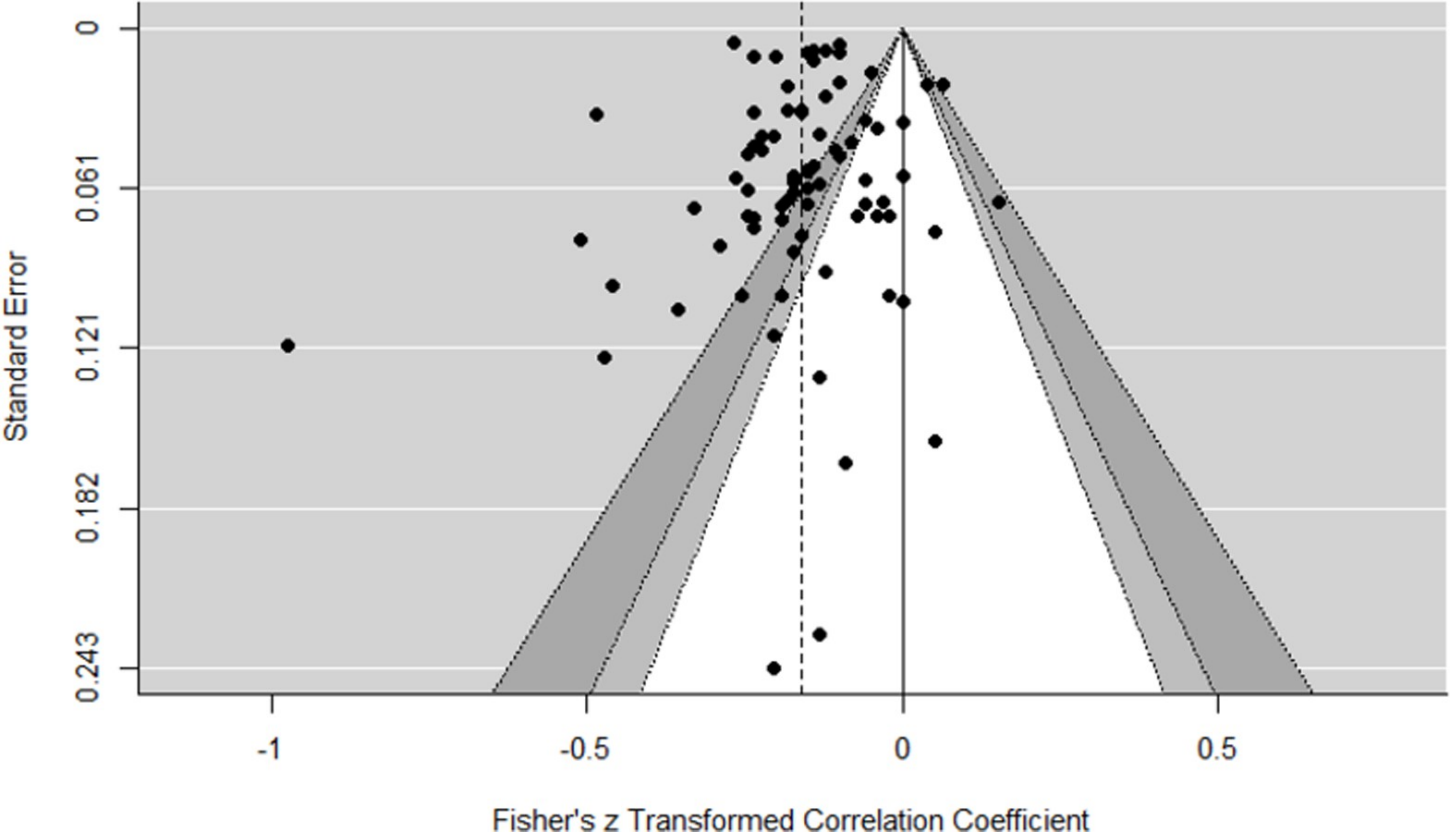
Contour-enhanced funnel plot of published effect sizes (k = 88). The dashed line represents the summary effect estimate; the vertical line represents the null effect; confidence lines delimit non-significance of effect sizes within (ps: white = .10, light grey = .05, dark grey = .01).

Only published study effects were included in all dissemination bias analyses (*k* = 88). Visual inspection of the funnel plot (Fig 8) did not show any obvious signs for asymmetry, excepting one outlier that had been already identified in our influence diagnostics. Formal tests of funnel plot asymmetry were consistent with this assessment. Neither Trim-and-Fill analysis (no indication of missing studies on the right side of the funnel plot), Sterne and Egger`s regression test (*Z* = -1.00, *p* = .32), nor the rank correlation method (τ = -0.121, *p* = .10) yielded any evidence for funnel plot asymmetry.

In order to test for excessive significance, the average power based on the observed study effect was calculated (within-study average power = 63%). Consequently, the number of expected significant studies in the hypothesis-conforming direction was 66. There were less studies with a significant outcome observed than what would have been expected based on our power calculations, thus indicating no evidence for bias.

**Fig 9:**
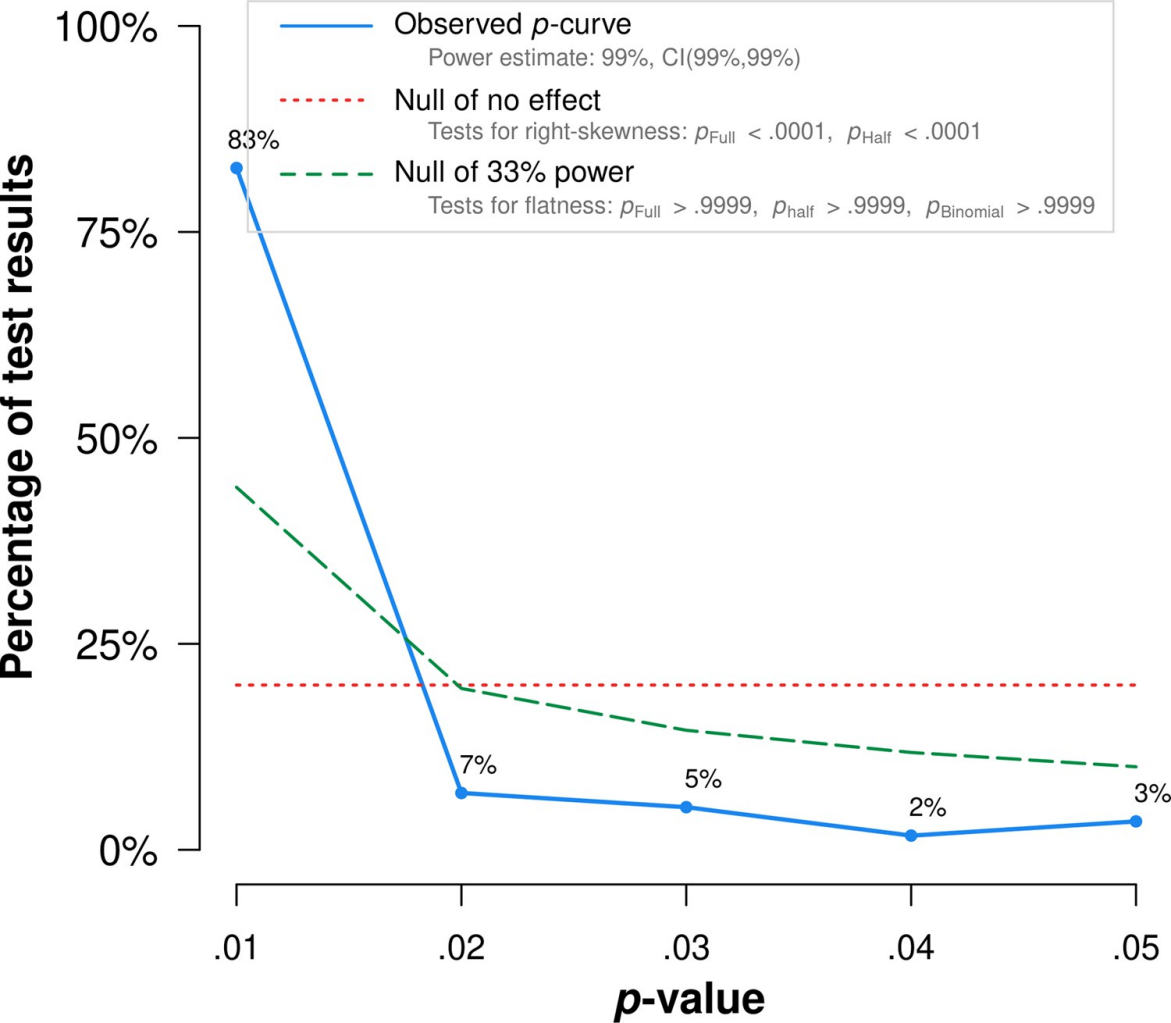
p-curve. Distribution of significant (α < .05) p-values of published findings.

Both *p*-curve and *p*-uniform analyses were based on 55 published significant studies. We observed a right-skewed *p*-curve (Fig 9) with more small (i.e., values that were close to *p* = .01) than large *p*-values (i.e., values that were close to *p* = .05), indicating that the present body of research holds empirical evidence and the extent of *p*-hacking is negligible. Broadly in line with our findings from our random-effects analysis, *p*-curve yielded an effect size estimate of *r* = -.16.

In our *p*-uniform analyses the *pp*-value distribution of the fixed-effect estimate did not differ substantially from the uniform distribution (*p* = .67), indicating no evidence for dissemination bias. The *p*-uniform effect estimate was similar to the estimation based on our random-effects analysis and *p-*curve *r* = -.17 (95% CI [-.20; -.15]; *p* < .001). The slightly larger effect estimates of *p*-curve and *p*-uniform compared to the random-effects analysis was to be expected and is most likely due to the typically observed effect overestimations of these two methods in presence of non-trivial between-studies heterogeneity [52].

The *p*-uniform*-based estimate was similar to the *p*-curve and *p*-uniform estimates, yielding *r* = -.16. Consequently, all our used dissemination bias detection and effect estimation methods convergently pointed towards negligible effects of dissemination bias in our present meta-analysis.

#### Time trends

**Fig 10:**
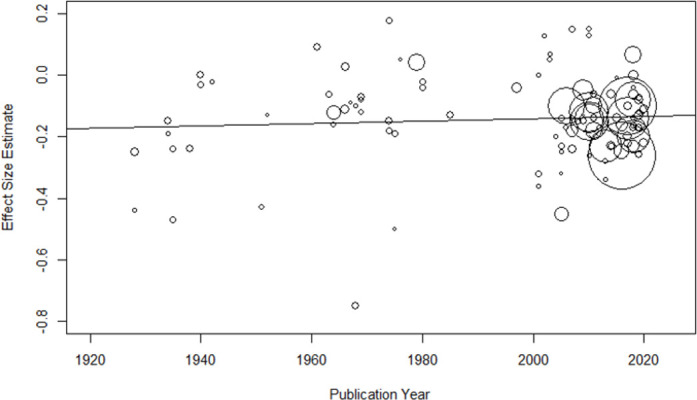
Cross-temporal meta-regression. Symbol sizes are varied according to the relative study weights.

A weighted meta-regression of effect sizes on study publication years showed neither effect strength declines over time in all (*b* < 0.001; *Q* = 0.54, *R*^2^ < .01, *p* = .46) or only published effect sizes (i.e., published effects should be more prone to show a decline effect; *k* = 88; *b* < 0.001; *Q* = 0.39, *R*^2^ < .01, *p* = .63; Fig 10). Interestingly, the signs of the regression coefficients were indicative of increases in effect strength, thus ruling decline effects in the present study out altogether [35].

#### Primary study quality assessments

A simple regression of effect sizes on study quality showed no significant effect (*b* = -0.004, *R*^2^ < .01, *p* = .75). We repeated this analysis in a precision-weighted meta-regression and obtained virtually identical results (*b* > -0.001, *R*^2^ < .01, *p* = .93), thus corroborating that study quality did not affect the observed associations.

## Mediation analyses

### Education

We conducted mediation analyses to examine potentially mediating effects of education on the intelligence and religiosity link. College samples were excluded from these analyses, because they necessarily comprise participants with identical highest educational qualifications. We used correlations of either type of religiosity measure, but correlations with measures of beliefs were preferred over those measuring behaviors, when both correlations were provided.

Meta-analytic correlations revealed a significant negative association (*r* = -.17, 95% *CI* [-.20; -.13], *p* < .001) that remained virtually identical in terms of strength when education was controlled for in partial correlations (*r* = -.15, 95% *CI* [-.20; -.11], *p* < .001; partial correlations of primary studies are provided in the S6 Appendix). This indicates no meaningful effects of education on the intelligence and religiosity association. In contrast, the significant education and religiosity link (*r* = -.06, 95% *CI* [-.09; -.02], *p* = .003) was virtually reduced to zero and failed to reach nominal statistical significance when intelligence was controlled for (*r* = -.01, 95% *CI* [-.05; -.02], *p* = .44; partial correlations of primary studies are provided in the S7 Appendix), thus indicating a full mediation by intelligence.

**Fig 11:**
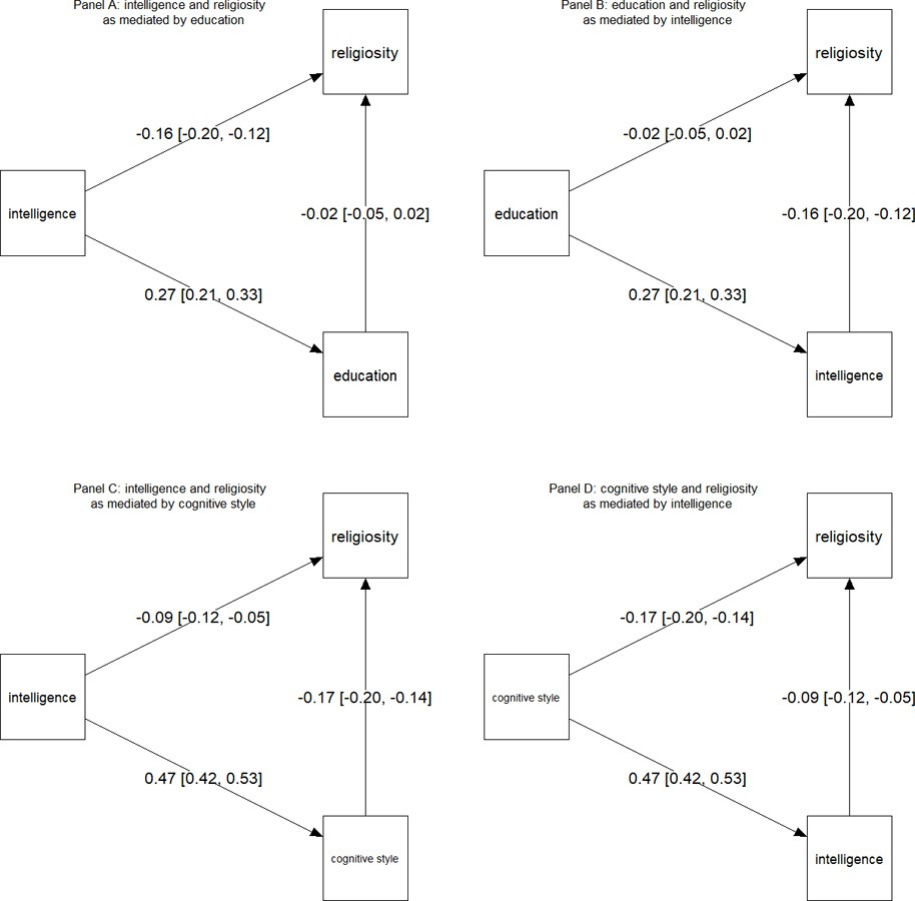
Mediation analyses.

These results were corroborated by formal meta-analytical mediation analyses. The (mediated) indirect association between intelligence and religiosity did not yield significant results (*b* = -0.014; 95% *CI* [-0.005; 0.006]). The direct path from intelligence to religiosity remained virtually identical when education was accounted for (*b*s = -0.17 vs. -0.16, respectively; Fig 11, Panel A), indicating no mediating influences of education. When we assumed intelligence as the mediating variable on the path of education on intelligence, we observed a significant negative indirect effect (*b* = -0.043; 95% *CI* [-0.058;.-0.030]) and the direct effect of education on religiosity disappeared when accounting for intelligence, thus indicating a full mediation of this path by intelligence (*b*s = -0.06 vs. -0.02; Fig 11, Panel B).

### Cognitive style

In another mediation model, we examined potentially mediating effects of cognitive styles (i.e., analytic vs. intuitive styles) on the intelligence and religiosity association. Here, our analytic approach was the same as described above (in these analyses, we also included data of college samples). The significant intelligence and religiosity link (*r* = -.17, 95% *CI* [-.20; -.14], *p* < .001) was considerably reduced in strength, when cognitive style was controlled for (*r* = .09, 95% *CI* [-.12; -.06], *p* < .001), thus indicating a partial mediation effect. Similarly, the association of analytic style and religiosity was significant (*r* = -.21; 95% *CI* [-.24; -.18], *p* < .001), but substantially reduced when intelligence was controlled for in partial correlations (*r* = -.15, 95% *CI* [-.19; -.11], *p* < .001).

Formal tests in meta-analytical mediation models once more supported our findings from the correlation analyses. Both indirect effects were negative as well as significant (*b* = -0.080; 95% *CI* [-0.100;.-0.063] and *b* = -0.042; 95% *CI* [-0.059;.-0.026], respectively) and both direct paths from intelligence and cognitive style on religiosity were partially mediated by cognitive style (*b*s = -0.17 vs. -0.09, Fig 11, Panel C) and intelligence (*b*s = -0.15 vs. -0.09; Fig 11, Panel D).

## Discussion

In the present meta-analysis, we provide evidence for robust, albeit small, associations between intelligence and religiosity. Convergent results from a large number of reasonable specifications indicate that these effects generalize and remain robust even when accounting for different moderators, with some allowance to be made for varying effect strengths. The intelligence and religiosity link appears to be therefore virtually ubiquitous, although the presently identified differences in strength present several points of interest, as we discuss below.

First, the intelligence and religiosity link appeared to be stronger when psychometric intelligence was assessed compared to less salient proxies of intelligence such as GPA. This seems reasonable because achievement measures such as GPA are noisy measures of cognitive abilities. School grades are well-known to be driven to a considerable extent by factors such as motivation [53], school environment [54], or socioeconomic status [55], consequently yielding a performance indicator that represents a suboptimal measure of competency. The observation that religiosity correlations with academic achievement measures conform in terms of the sign to correlations with intelligence once more shows the robustness of this link.

Second, the type of religiosity measure that had been used in primary studies appeared to moderate the observed associations, although nominal significance was marginally not reached in our subgroup analysis. All presently included studies used self-reports to assess religiosity, but those that were framed on religious beliefs yielded larger correlations than religious behaviors. This may be attributed to religious behavior (e.g., praying, church attendance, belonging to a denomination or not) being a poorer measure of an individual’s religiosity than self-reported beliefs, because behaviors may be motivated by the desire to conform to an in-group instead of religiosity (i.e., religious group-membership and its related behavioral expectations) [56]. These results reemphasize the importance of distinguishing between intrinsic (i.e., beliefs) and extrinsic motivation (i.e., behavior) when assessing religiosity [57].

Third, the educational status of samples moderated the strength of intelligence and religiosity associations. Correlations of non-college samples were substantially stronger than those of college samples, but differences disappeared when range restriction was accounted for. This means, that previously observed lower associations in better educated samples [6] are most likely an artifact of homogeneous (attenuated) samples.

Although college and non-college samples appear to show virtually identical associations, effects of pre-college samples were substantially smaller, indicating only a trivial intelligence and religiosity association in children and adolescents. A possible explanation for this observation could be that religious worldviews become only fully developed in more mature ages [58]. Although due to the correlational nature of the synthesized studies, causality cannot be inferred, it may speculated that intelligence (or at least one of its covariates) is responsible for the development of certain religious beliefs, rather than the other way around. Longitudinal data are needed to test these ideas.

Fourth, sex had been previously observed to be a potentially influential variable with women showing typically larger effects than men [5], although not all analytic approaches supported this interpretation [6,7]. Our results mirror these inconsistent findings, with continuous assessments indicating larger effects for men, but categorical assessments indicating larger effects for women. Considering prior evidence of virtually nill-effects of within-studies sex differences [6,7], this means most likely that sex does not play a meaningful role for intelligence and religiosity associations altogether.

Fifth, we did not find significant changes in study effect strengths over time, thus contrasting findings of a previous systematic review [5]. There was no evidence for dissemination bias that may have confounded the intelligence and religiosity link. Direct comparisons of published and unpublished studies neither yielded significant nor meaningful differences between subset summary effects. Moreover, application of several standard and modern dissemination bias detection methods as well as novel effect estimation methods showed convergent evidence for the validity and the robustness of the observed summary effect. This means that, although the strength of the intelligence and religiosity association appears to be differentiated over a number of moderators, the observed negative link is not confounded by research process-related artifacts and generalizes over subgroups. Importantly, primary study quality did not emerge as a meaningful predictor for the intelligence and religiosity link either, thus largely ruling out potential effects of primary study design features.

### Education

Based on evidence from longitudinal data, it has been hypothesized, that between-individual differences in education rather than intelligence are most likely the drivers of the intelligence and religiosity link [59,60]. We found that education does not mediate the intelligence and religiosity link, thus contrasting this idea and an earlier meta-analytical account [5]. Interestingly though, we observed a full mediation of intelligence on the education and religiosity association, indicating that intelligence but not education is a more likely driver of intelligence and thus corroborating findings from a previous meta-analysis [7].

### Cognitive style

Although the current meta-analytic data do not allow drawing conclusions about causal inferences, it should be acknowledged, that the available evidence indicates intelligence impacting religiosity rather than the other way around. Whilst cognitive ability can be measured quite reliably at an early age and is a good predictor of intelligence in adulthood [55], religious involvement is less stable [61] and early assessed religiosity is a weak predictor of later religiosity [58]. In longitudinal studies [62–65], intelligence was measured several years before religiosity. In a previous review [6], summarizing these results led to a negative correlation of intelligence and religiosity when considering only belief-based measures, supporting a model in which intelligence drives religiosity.

On the whole, several plausible causes for a negative association of intelligence and religiosity have been suggested [6]. In the present meta-analysis, the role of cognitive style in the context of single and dual-process models of the mind is of particular interest, because we were able to empirically examine effects of analytic and intuitive styles on the intelligence and religiosity link.

According to the dual-process model of the mind [66,67] there are two different systems of information processing. One outputs intuitive judgments, because it is responsible for automatic information processing. The other one is responsible for systematic, analytic information processing. It has been shown that individuals differ in their tendencies to rely on one of these processing styles [67]. From this perspective, it could be argued that individuals that use a more intuitive (and therefore experiential) and a less analytic (and therefore rational) information processing style may be more accessible to religious ideals. This interpretation is supported by the observations that individuals that reported stronger beliefs in God showed lower analytical processing styles [68], whilst more intelligent people were found to prefer analytical over intuitive processing [69]. Our results in terms of mediating influences of cognitive style on the religiosity and intelligence association are consistent with this idea.

However, recent studies promote single-process models where analytic and intuitive cognitive styles represent the extremes on a continuum, thus adopting a dimensional rather than a categorical view of cognitive styles (for an overview, see [67]). Arguably, the observed partial cognitive style mediation of the intelligence and religiosity association may be seen to fit better to a single process rather than a dual-process model, because the cognitive style variables were treated as a dimensional rather than a categorical concept in primary studies and our meta-analysis.

### Limitations

Some limitations of the present meta-analysis need to be acknowledged. It would have been desirable to investigate if belongingness to different religious denominations influences the intelligence and religiosity association. For instance, Catholicism or Judaism are characterized by a strong embeddedness of believers in their community. Common religious practices and rituals including other people are an inherent part of religious identity [70]. American Protestantism on the other hand is more individualistic. These differences give reason to assume that the stronger negative association of intelligence and religious beliefs (in contrast to religious behavior) may be most pronounced for American Protestants [6], but perhaps less so for other denominations. Unfortunately, due to insufficient reporting in primary studies, potential moderating effects of religious affiliations could not be assessed. In a similar vein, the predominant part of included studies was conducted in Western countries, especially in the United States, therefore limiting the generalizability of our findings to Western contexts.

Moreover, it was not possible to assess potential effects of conceptual differences between spirituality and religiosity in the present study. For example, as individuals age, religiosity seems to increase stronger in Catholics than in Protestants [66] which may be related with a greater social embeddedness and therefore extrinsic motives to turn to religion. Such extrinsic reasons for religious behaviors are an important differentiating factor of religion and spirituality. It needs to be acknowledged that these terms represent conceptually non-identical constructs that, however, typically largely empirically overlap [71].

Because of sometimes uncertain dimensionality of assessment instruments and certain suboptimal methodical features of primary studies (e.g. single item assessments asking about beliefs in supernatural agents [72]), correlations of IQ with religiosity can be expected to be confounded by certain facets of spirituality in the present study. Although it cannot be ruled out that this ambiguity may have introduced some statistical noise into the present data, the considerable empirical overlap of these two constructs should largely alleviate concerns about the robustness of our results. In fact, it speaks even more for the robustness of our findings, that even in presence of these potential confounders, our results convergently indicated meaningful negative associations between intelligence and religiosity.

## Conclusion

The results of the present meta-analysis demonstrate a clear–albeit small–and almost ubiquitous negative association of religiosity with intelligence. This negative link was most pronounced when results of psychometric intelligence tests were correlated with self-reported beliefs and seemed to be strongest in members of the adult general population. In all, the convergent evidence from more standard and several modern approaches to meta-analyses, such as combinatorial, multiverse, and specification-curve analyses, as well as results from a large number of bias assessment methods indicated a remarkable robustness of this effect that generalizes in direction and meaningfulness (although not strength) across moderators.

## Supporting information

S1 Appendix Reference list of included studies. (DOCX)

S2 Appendix Adapted Newcastle Ottawa Scale and primary study quality ratings. (DOCX)

S3 Appendix R-code for all analyses. (TXT)

S4 Appendix PRISMA checklist. (DOCX)

S5 Appendix Influence diagnostics for correlations of intelligence and religiosity. (PDF)

S6 Appendix Zero-order and partial correlations among intelligence, religiosity, and education. (DOCX)

S7 Appendix Zero-order and partial correlations among intelligence, religiosity, and analytic style. (DOCX)
