# Supplementary material for: Meta-analyzing intelligence and religiosity associations: Evidence from the multiverse
Source: PLoS One. 2022 Feb 11;17(2):e0262699. doi: 10.1371/journal.pone.0262699 (PMC8836311; doi:10.1371/journal.pone.0262699)
Supplement: S1 Appendix — (DOCX) [file pone.0262699.s001.docx]

**S1 Appendix. Reference list of included studies.**

Bender, I. E. (1968). A longitudinal study of church attenders and nonattenders. *Journal for the Scientific Study of Religion, 7,* 230-237. doi:10.2307/1384630

Bertsch, S., & Pesta, B. J. (2009). The wonderlic personnel test and elementary cognitive tasks as predictors of religious sectarianism, scriptural acceptance and religious questioning. *Intelligence, 37,* 231-237. doi:10.1016/j.intell.2008.10.003

Betsch, T., Aßmann, L., & Glöckner, A. (2020). Paranormal beliefs and individual differences: story seeking without reasoned review. *Heliyon, 6*, 1-8.

Blanchard-Fields, F., Hertzog, C., Stein, R., & Pak, R. (2001). Beyond a stereotyped view of older adults’ traditional family values. *Psychology and Aging, 16,* 483-496. doi:10.1037/0882- 7974.16.3.483

Bloodgood, J. M., Turnley, W. H., & Mudrack, P. (2008). The influence of ethics instruction, religiosity, and intelligence on cheating behavior. *Journal of Business Ethics, 82,* 557-571. doi:10.1007/s10551-007-9576-0

Boazman, J. K. (2010). *Well-being and academic success in gifted college students: early- College entrants and honors college students.* Dissertation Thesis, University of North Texas.

Bronstein, M. V., Pennycook, G., Bear, A., Rand, D. G., & Cannon, T. D. (2019). Belief in fake news is associated with delusionality, dogmatism, religious fundamentalism, and reduced analytic thinking. *Journal of Applied Research in Memory and Cognition, 8*, 108-117.

Brown, D. G., & Lowe, W. L. (1951). Religious beliefs and personality characteristics of college students. *The Journal of Social Psychology, 33,* 103-129. doi:10.1080/00224545.1951 .9921803

Carlson, H. B. (1934). Attitudes of undergraduate students. *The Journal of Social Psychology, 5,* 202-213. doi:10.1080/00224 545.1934.9919448

Carothers, S. S., Borkowski, J. G., Burke Lefever, J., & Whitman, T. L. (2005). Religiosity and the socioemotional adjustment of adolescent mothers and their children. *Journal of Family Psychology, 19,* 263-275. doi:10.1037/0893-3200.19.2.263

Čavojová, V., Secară, E., Jurkoviĉ, M., & Šrol, J. (2018). Reception and willingness to share pseudo-profound bullshit and their relation to other epistemically suspect beliefs and cognitive ability in Slovakia and Romania. *Applied Cognitive Psychology, 33*, 299-311.

Čavojová, V., Šrol, J., & Jurkoviĉ, M. (2019). Why should we try to think like scientists? Scientific reasoning and susceptibility to epistemicalls suspect beliefs and cognitive biases. *Applied Cognitive Psychology, 34*, 85-95.

Ciesielski-Kaiser, H. A. (2005). *Religious identity development and personality* (Unpublished doctoral dissertation). Southern Illinois University Carbondale, Carbondale.

Clark, R. (2004). Religiousness, spirituality, and IQ: are they linked? *Explorations: An Undergraduate Research Journal,* 35-46.

Corey, S. M. (1940). Changes in the opinions of female students after one year at a university. *The Journal of Social Psychology, 11,* 341-351. doi:10.1080/00224545.1940.9918754

Cottone, J., Drucker, P., & Javier, R. A. (2007). Predictors of moral reasoning: Components of executive functioning and aspects of religiosity. *Journal for the Scientific Study of Religion, 46,* 37-53. doi:10.1111/j.1468-5906.2007.00339.x

Crossman, A. M. (2001). *Predicting suggestability: The role of individual differences and socialization* (Unpublished doctoral dissertation). Cornell University, Ithaca, NY.

Daws, R. R., & Hampshire, A. (2017). The negative relationship between reasoning and religiosity is underpinned by a bias for intuitive responses specifically when intuition and logic are in conflict. *Frontiers in Psychology, 8,* Article 2191. doi:10.3389/ fpsyg.2017.02191

Deptula, D. P., Henry, D. B., Shoeny, M. E., & Slavick, J. T. (2006). Adolescent sexual behavior and attitudes: A costs and benefits approach. *Journal of Adolescent Health, 38,* 35-43. doi:10.1016/j.jadohealth.2004.08.026

Dodrill, C. B. (1976). Brain functions of Christians and non-Christians. *Journal of Psychology & Theology, 4,* 280-285.

Dreger, R. M. (1952). Some personality correlates of religious attitudes, as determined by projective techniques. *Psychological Monographs: General and Applied, 66,* 1-18. doi:10.1037/ h0093604

Drewelies, J., Deeg, D. J. H., Huisman, M., & Gerstorf, D. (2018). Perceived constrains in late midlife: cohort differences in the Longitudinal Aging Study Amsterdam (LASA). *Psychology and Aging, 33*, 754-768.

Erlandsson, A., Nilsson, A., Tinghög, G., & Västfjäll, D. (2018). Bullshit-sensitivity predicts prosocial behavior. *PloS ONE, 13*, e0201474.

Feather, N. T. (1964). Acceptance and rejection of arguments in relation to attitude strength, critical ability, and intolerance of inconsistency. *Journal of Abnormal and Social Psychology, 69*, 127-136. doi:10.1037/h0046290

Feather, N. T. (1967). Evaluation of religious and neutral arguments in religious and atheist student groups. *Australian Journal of Psychology, 19,* 3-12. doi:10.1080/00049536708255556

Foong, H. F., Hamid, T. A., Ibrahim, R., & Haron, S. A. (2018). Moderating effect of intrinsic religiosity on the relationship between depression and cognitive function among community-dwelling older adults. *Aging & Mental Health, 22*, 483-488.

Foy, D. W. (1975). *A study of the relationship between intelligence, religiosity and locus of control* (Unpublished doctoral dissertation). University of Southern Mississippi, Hattiesburg.

Francis, L. J. (1979). School influence and pupil attitude towards religion. *British Journal of Educational Psychology, 49,* 107- 123. doi:10.1111/j.2044-8279.1979.tb02405.x

Francis, L. J., Pearson, P. R., & Stubbs, M. T. (1985). Personality and religion among low ability children in residential special schools. *The British Journal of Mental Subnormality, 31,* 41-45.

Francis, L. J. (1997). Personal and social correlates of the “closed mind” among 16 year old adolescents in England. *Educational Studies, 23,* 429-437. doi:10.1080/0305569970230308

Francis, L. J. (1998). The relationship between intelligence and religiosity among 15-16 year olds. *Mental Health, Religion and Culture, 1,* 185-196. doi:10.1080/13674679808406508

Franzblau, A. N. (1934). *Religious belief and character among Jewish adolescents.* New York, NY: Teachers College, Columbia University Bureau of Publications.

Furnham, A., & Grover, S. (2020). Correlates of self-estimated intelligence. *Journal of Intelligence, 8*, 6.

Ganzach, Y., & Gotlibovski, C. (2013). Intelligence and religiosity: within families and over time. *Intelligence, 41,* 546-552.

Gilliland, A. R. (1940). The attitude of college students toward God and the church. *The Journal of Social Psychology, 11,* 11- 18. doi:10.1080/00224545.1940.9918729

Gragg, D. B. (1942). Religious attitudes of denominational college students. *The Journal of Social Psychology, 15,* 245-254. doi:1 0.1080/00224545.1942.9921534

Hadden, J. K. (1963). An analysis of some factors associated with religion and political affiliation in a college population. *Journal for the Scientific Study of Religion, 2,* 209-216. doi:10.2307/1385072

Hartman, R. O., Dieckmann, N. F., Sprenger, A. M., Stastny, B. J., & DeMarree, K. G. (2017). Modeling attitudes toward science: Development and validation of the Credibility of Science Scale. *Basic and Applied Social Psychology, 39,* 358-371. doi: 10.1080/01973533.2017.1372284

Heaven, P. C. L., Ciarrochi, J., & Leeson, P. (2011). Cognitive ability, right-wing authoritarianism, and social dominance orientation: A five-year longitudinal study amongst adolescents. *Intelligence, 39,* 15-21. doi:10.1016/j.intell.2010.12.001

Hergovich, A., & Arendasy, M. (2005). Critical thinking ability and belief in the paranormal. *Personality and Individual Differences, 38,* 1805-1812.

Hoge, D. R. (1969). *College student’s religion: A study of trends in attitudes and behavior* (Unpublished doctoral dissertation). Harvard University, Cambridge, MA.

Horowitz, J. L., & Garber, J. (2003). Relation of intelligence and religiosity to depressive disorders in offspring of depressed and nondepressed mothers. *Journal of the American Academy of Child & Adolescent Psychiatry, 42,* 578-586. doi:10.1097/01. CHI.0000046831.09750.03

Howells, T. H. (1928). A comparative study of those who accept as against those who reject religious authority. *University of Iowa Studies in Character, 2*, 1-80.

Inzlicht, M., McGregor, I., Hirsh, J. B., & Nash, K. (2009). Neural markers of religious conviction. *Psychological Science, 20,* 385-392. doi:10.1111/j.1467-9280.2009.02305.x

Jones, V. (1938). Attitudes of college students and the changes in such attitudes during four years in college. *Journal of Educational Psychology, 29,* 14-25. doi:10.1037/h0055012

Kahoe, R. D. (1974). Personality and achievement correlates of intrinsic and extrinsic religious orientations. *Journal of Personality and Social Psychology,* *29,* 812-818.

Kanazawa, S. (2010a). Why liberals and atheists are more intelligent. *Social Psychology Quarterly, 73,* 33-57. doi:10.1177/019027 2510361602

Kirkegaard, E. O. W., & Bjerrekaer, J. D. (2016). *The OKCupid dataset: A very large public dataset of dating site users*. Open Differential Psychology. Advance online publication. doi:10.26775/ODP.2016.11.03

Kosa, J., & Schommer, C. O. (1961). Religious participation, religious knowledge, and scholastic aptitude: An empirical study. *Journal for the Scientific Study of Religion, 1,* 88-97. doi:10.2307/1385179

Leonard, C. A. (2018). *Fallacious beliefs: gambling specific and belief in the paranormal.* Dissertation Thesis, University of Lethbridge, Canada.

Lewis, G. J., Ritchie, S. J., & Bates, T. C. (2011). The relationship between intelligence and multiple domains of religious belief: Evidence from a large adult US sample. *Intelligence, 39,* 468- 472. doi:10.1016/j.intell.2011.08.002

Łowicki, P., Zajenkowski, M., & van der Linden D. (2020) The interplay between cognitive intelligence, ability, emotional intelligence and religiosity. *Journal of Religion and Health, 59,* 2556-2576.

McCullough, M. E., Enders, C. K., Brion, S. L., & Jain, A. R. (2005). The varieties of religious development in adulthood: A longitudinal investigation of religion and rational choice. *Journal of Personality and Social Psychology, 89,* 78-89. doi:10.1037/0022-3514.89.1.78

Nilsson, A., Erlandsson, A., & Västfjäll, D. (2019). The complex relation between receptivity to pseudo-profound bullshit and political ideology. *Personality and Social Psychology Bulletin, 45,* 1440-1454.

Nokelainen, P., & Tirri, K. (2010). Role of motivation in the moral and religious judgment of mathematically gifted adolescents. *High Ability Studies, 21,* 101-116. doi:10.1080/13598139.201 0.525343

Nyborg, H. (2009). The intelligence-religiosity nexus: A representative study of white adolescent Americans. *Intelligence, 37,* 81-93. doi:10.1016/j.intell.2008.08.003

Patel, N., Baker, S. G., & Scherer, L. D. (2019). Evaluating the cognitive reflection test as a measure of intuition/ reflection, numeracy, and insight problem solving, and the implications for understanding real-world judgments and beliefs. *Journal of Experimental Psychology: General, 148,* 2129-2153.

Pennycook, G., Cheyne, J. A., Barr, N., Koehler, D. J., & Fugelsang, J. A. (2014a). The role of analytic thinking in moral judgments and values. *Thinking & Reasoning, 20,* 188-214.

Pennycook, G., Cheyne, J. A., Barr, N., Koehler, D. J., & Fugelsang, J. A. (2014b). Cognitive style and religiosity: The role of conflict detection. *Memory & Cognition, 42,* 1-10. doi:10.3758/s13421-013-0340-7

Pennycook, G., Cheyne, J. A., Koehler, P. J., & Fugelsang, J. A. (2013). Belief bias during Reasoning among religious believers and skeptics. *Psychological Bulletin Review, 20,* 806-811.

Pennycook, G., Cheyne, J. A., Seli, P., Koehler, D. J., & Fugelsang, J. A. (2012). Analytic cognitive style predicts religious and paranormal belief. *Cognition, 123,* 335-346. doi:10.1016/j. cognition.2012.03.003

Pennycook, G., Ross, R. M., Koehler, D. J., & Fugelsang, J. A. (2016). Atheists and agnostics are more reflective than religious believers: Four empirical studies and a meta-analysis. *PLoS ONE, 11,* e0153039. doi:10.1371/journal.pone.0153039

Perales, F. (2018). The cognitive roots of prejudice towards samesex couples: An analysis of an Australian national sample. *Intelligence, 68,* 117-127. doi:10.1016/j.intell.2018.03.012

Pollet, E., & Schnell, T. (2017). Brilliant: But what for? Meaning and subjective well-being in the lives of intellectually gifted and academically high-achieving adults. *Journal of Happiness Studies, 18,* 1459-1484. doi:10.1007/s10902-016-9783-4

Poythress, N. G. (1975). Literal, antiliteral, and mythological religious orientations. *Journal for the Scientific Study of Religion, 14,* 271-284. doi:10.2307/1384909

Räsänen, A., Tirri, K., & Nokelainen, P. (2006). *The moral and religious reasoning of gifted adolescence.* In K. Tirri (Ed.), Nordic perspectives on religion, spirituality and identity (pp. 97-111). Helsinki, Finland: University of Helsinki.

Raman, J. R. (2010). *Correlates of tertiary student life satisfaction.* Master`s Thesis, University of Waikato.

Razmyar, S., & Reeve, C. L. (2013). Individual differences in religiosity as a function of cognitive ability and cognitive style. *Intelligence, 41,* 667-673.

Ritchie, S. J., Gow, A. J., & Deary, I. J. (2014). Religiosity is negatively associated with later-life intelligence, but not with age-related cognitive decline. *Intelligence, 46,* 9-17.

Ross, R. M. (2015). *Cognitive and evolutionary foundations of culture and belief* (PhD thesis). Macquarie University, Sydney, New South Wales, Australia.

Sacher, S. G. (2015). *An investigation of the link between spirituality and intelligence.* Master`s Degree, University of Saskatchewan.

Salter, C. A., & Routledge, L. M. (1974). Intelligence and belief in the supernatural. *Psychological Reports, 34,* 299-302. doi:10.2466/pr0.1974.34.1.299

Saribay, S. A., & Yilmaz, O. (2017). Analytic cognitive style and cognitive ability differentially predict religiosity and social conservatism. *Personality and Individual Differences, 114,* 24-29.

Saroglou, V., & Fiasse, L. (2003). Birth order, personality, and religion: A study among young adults from a three-sibling family. *Personality and Individual Differences, 35,* 19-29. doi:10.1016/S0191-8869(02)00137-X

Saroglou, V., & Scariot, C. (2002). Humor Styles Questionnaire: Personality and educational correlates in Belgian high school and college students. *European Journal of Personality, 16,* 43- 54. doi:10.1002/per.430

Shenhav, A., Rand, D. G., & Greene, J. D. (2012). Divine intuition: Cognitive style influences belief in God. *Journal of Experimental Psychology: General, 141,* 423-428. doi:10.1037/ a0025391

Sherkat, D. E. (2010). Religion and verbal ability. *Social Science Research, 39,* 2-13. doi:10.1016/j.ssresearch.2009.05.007

Sherkat, D. E. (2011). Religion and scientific literacy in the United States. *Social Science Quarterly, 92,* 1134-1150. doi:10.1111/ j.1540-6237.2011.00811.x

Sinclair, R. D. (1928). A comparative study of those who report the experience of the divine presence and those who do not. *University of Iowa Studies in Character, 2,* 1-62.

Southern, M. L., & Plant, W. T. (1968). Personality characteristics of very bright adults. *The Journal of Social Psychology, 75,* 119-126.

Stagnaro, M. N., Ross, R. M., Pennycook, G., & Rand, D. G. (2019). Cross-cultural support for a link between analytic thinking and disbelief in God: evidence from India and the United Kingdom. *Judgment and Decision Making, 14*, 179-186.

Ståhl, T., & van Prooijen, J. (2018). Epistemic rationality: skepticism toward unfounded beliefs requires sufficient cognitive ability and motivation to be rational. *Personality and Individual Differences, 122,* 155-163.

Stankov, L., & Lee, J. (2018). Conservative syndrome and the understanding of negative correlations between religiosity and cognitive abilities. *Personality and Individual Differences, 131,* 21-25.

Stanovich, K. E., & West, R. F. (2007). Natural myside bias is independent of cognitive ability. *Thinking & Reasoning, 13,* 225-247. doi:10.1080/13546780600780796

Strimaitis, J. L. (2018). *Conditional reflection and the religion reflection scale: how familiarity with different domains influences cognitive reflection.* Master`s Thesis, *U*niversity of Tennesee at Chattanooga.

Symington, T. A. (1935). *Religious liberals and conservatives.* New York, NY: Teachers College, Columbia University Bureau of Publications

Szobot, C. M., Rohde, L. A., Bukstein, O., Molina, B. S. G., Martins, C., Ruaro, P., & Pechansky, F. (2007). Is attentiondeficit/hyperactivity disorder associated with illicit substance use disorders in male adolescents? A community-based casecontrol study. *Addiction, 102,* 1122-1130. doi:10.1111/j.1360- 0443.2007.01850.x

Turner, E. B. (1980). General cognitive ability and religious attitudes in two school systems. *The British Journal of Religious Education, 2,* 136-141. doi:10.1080/0141620800020404

Verhage, F. (1964). Intelligence and religious persuasion. *Nederlands Tijdschrift voor de Psychologie en Haar Grensgebieden, 19,* 247-254.

Wahlig, E. L. (2005). *Health outcomes in multiple sclerosis: The role of beliefs and personality in cognitive function, depression, fatigue, and quality of life* (Unpublished doctoral dissertation). The State University of New York at Buffalo

Young, R. K., Dustin, D. S., & Holtzman, W. H. (1966). Change in attitude toward religion in a southern university. *Psychological Reports, 18,* 39-46. doi:10.2466/pr0.1966.18.1.39

Zuckerman, M., & McPhetres, J. (2016). Unpublished data.
