# Supplementary material for: Meta-analyzing intelligence and religiosity associations: Evidence from the multiverse
Source: PLoS One. 2022 Feb 11;17(2):e0262699. doi: 10.1371/journal.pone.0262699 (PMC8836311; doi:10.1371/journal.pone.0262699)
Supplement: S2 Appendix — (DOCX) [file pone.0262699.s002.docx]

S2 Appendix: Adapted Newcastle Ottawa Scale for primary study quality assessment

1. Representativeness of the sample:

a) Truly representative of the average in the target population. * (all subjects or random sampling)

b) Somewhat representative of the average in the target population. * (nonrandom sampling)

c) Selected group of users.

d) No description of the sampling strategy.

1. Sample size:

a) Justified and satisfactory. *

b) Not justified.

1. Non-respondents:

a) Comparability between respondents and non-respondents characteristics is established, and the response rate is satisfactory. *

b) The response rate is unsatisfactory, or the comparability between respondents and non-respondents is unsatisfactory.

c) No description of the response rate or the characteristics of the responders and the non-responders.

1. Ascertainment of the exposure (risk factor):

a) Validated measurement tool. **

b) Non-validated measurement tool, but the tool is available or described.*

c) No description of the measurement tool.

| Study | Selection 1 | Selection 2 | Selection 3 | Selection 4 | Score |
| --- | --- | --- | --- | --- | --- |
| Howells (1928) | n/a | n/a | n/a | n/a | n/a |
| Sinclair (1928) | n/a | n/a | n/a | n/a | n/a |
| Carlson (1934) | a | b | b | b | 2 |
| Franzblau (1934) | n/a | n/a | n/a | n/a | n/a |
| (1)Symington (1935) | n/a | n/a | n/a | n/a | n/a |
| (2)Symington (1935) | n/a | n/a | n/a | n/a | n/a |
| V.Jones (1938)^a^ | c | b | c | b | 1 |
| Corey (1940) | d | b | b | b | 1 |
| Gilliland (1940) | d | b | c | c | 0 |
| Gragg (1942) | b | b | c | b | 2 |
| D.G.Brown, Lowe (1951) | b | b | c | b | 2 |
| Dreger (1952) | c | b | b | a | 2 |
| Kosa, Schommer (1961) | d | b | c | b | 1 |
| Hadden (1963) | a | b | b | b | 2 |
| Feather (1964) | n/a | n/a | n/a | n/a | n/a |
| Verhage (1964) | n/a | n/a | n/a | n/a | n/a |
| (1)Young, Dustin, Holtzman (1966) | a | b | b | b | 2 |
| (2)Young, Dustin, Holtzman (1966) | a | b | b | b | 2 |
| Feather (1967) | n/a | n/a | n/a | n/a | n/a |
| Southern, Plant (1968) | n/a | n/a | n/a | n/a | n/a |
| Bender (1968) | d | b | c | b | 1 |
| (1)Hoge (1969) | n/a | n/a | n/a | n/a | n/a |
| (2)Hoge (1969) | n/a | n/a | n/a | n/a | n/a |
| (3)Hoge (1969) | n/a | n/a | n/a | n/a | n/a |
| (1)Salter, Routledge (1974) | b | b | b | b | 2 |
| (2)Salter, Routledge (1974) | b | b | b | b | 2 |
| Kahoe (1974) | c | b | c | b | 1 |
| Foy (1975) | n/a | n/a | n/a | n/a | n/a |
| Poythress (1975) | c | b | b | b | 1 |
| Dodrill (1976)^a^ | c | b | c | a | 2 |
| Francis (1979) | n/a | n/a | n/a | n/a | n/a |
| (1)Turner (1980) | n/a | n/a | n/a | n/a | n/a |
| (2)Turner (1980) | n/a | n/a | n/a | n/a | n/a |
| Francis, Pearson, Stubbs (1985) | d | b | c | c | 0 |
| Francis;Francis (1997) | d | b | c | a | 2 |
| Crossman (2001) | n/a | n/a | n/a | n/a | n/a |
| (1)Blanchard-Fields et al. (2001) | c | b | c | a | 2 |
| (2)Blanchard-Fields et al. (2001) | c | b | c | a | 2 |
| Saroglou, Scariot (2002) | c | b | c | b | 1 |
| Saroglou, Fiasse (2003) | d | b | c | b | 1 |
| Horowitz, Garber (2003) | c | b | c | a | 2 |
| Clark (2004) | c | b | c | a | 2 |
| Ciesielski-Kaiser (2005) | n/a | n/a | n/a | n/a | n/a |
| McCullough et al. (2005)^a^ | c | a | b | b | 2 |
| Carothers et al. (2005)^a^ | b | b | c | a | 3 |
| Hergovich, Arendasy (2005) | c | b | c | a | 2 |
| Wahling (2005) | n/a | n/a | n/a | n/a | n/a |
| Deptula et al. (2006)^a^ | a | a | c | a | 4 |
| Räsänen, Tirri, Nokelainen (2006) | n/a | n/a | n/a | n/a | n/a |
| Cottone, Drucker, Javier (2007) | b | a | b | a | 4 |
| (1)Stanovich, West (2007) | c b | b | c | b | 1 |
| (2)Stanovich, West (2007) | c | b | c | b | 1 |
| Szobot et al. (2007)^b^ | a | a | a | b | 4 |
| Bloodgood, Turnley, Mudrack (2008) | c | b | c | b | 1 |
| Bertsch, Pesta (2009) | c | b | c | a | 2 |
| Inzlicht et al. (2009) | c | b | c | a | 2 |
| Nyborg (2009) | b | a | b | b | 3 |
| (1)Kanazawa (2010) | b | b | b | a | 3 |
| (2)Kanazawa (2010) | b | b | b | b | 2 |
| Nokelainen, Tirri (2010) | c | b | c | a | 2 |
| Raman (2010) | c | a | c | a | 3 |
| (1)Boazman (2010)^a^ | c | b | b | b | 1 |
| (2)Boazman (2010)^a^ | c | b | b | b | 1 |
| Sherkat (2010) | a | a | c | a | 4 |
| Sherkat (2011) | a | a | b | b | 3 |
| Heaven, Ciarrochi and Leeson (2011) | b | b | b | b | 2 |
| Lewis, Ritchie, Bates (2011) | b | b | b | a | 3 |
| Shenav, Rand, Greene (2012) | d | b | c | a | 2 |
| (1)Pennycook et al. (2012) | d | b | b | a | 2 |
| (2)Pennycook et al. (2012) | d | b | b | a | 2 |
| Ganzach & Gotlibovski (2013)^a^ | b | a | b | a | 4 |
| Razmyar & Reeve (2013) | c | b | c | a | 2 |
| Pennycook et al. (2013) | d | b | c | a | 2 |
| Ritchie et al. (2014)^a^ | b | a | b | a | 4 |
| Pennycook et al. (2014a) | b | b | b | a | 3 |
| Pennycook et al. (2014b) | c | b | c | a | 2 |
| Ross (2015) | b | b | b | a | 3 |
| Sacher (2015) | c | b | b | a | 2 |
| Kirkegaard and Bjerrekaer (2016) | b | a | c | b | 3 |
| Pennycook, Ross, Koehler and Fugelsang (2016) | c | b | b | a | 2 |
| Zuckerman and McPhetres (2016) | n/a | n/a | n/a | n/a | n/a |
| (1) Daws and Hampshire (2017) | b | a | c | a | 4 |
| (2) Daws and Hampshire (2017) | b | a | c | a | 4 |
| Hartman, Dieckmann, Sprenger, Stastny and DeMarree (2017) | b | b | c | a | 3 |
| Pollet and Schnell (2017) | c | b | c | a | 2 |
| Saribay & Yilmaz (2017) | b | a | c | a | 4 |
| (1) Strimaitis (2018) | b | b | c | a | 3 |
| (2) Strimaitis (2018) | b | b | b | a | 3 |
| Stankov & Lee (2018) | b | a | c | a | 4 |
| (1) Drewelies, Deeg, Huisman, Gerstorf (2018)^a^ | a | a | b | a | 4 |
| (2) Drewelies, Deeg, Huisman, Gerstorf (2018)^a^ | a | a | b | a | 4 |
| Erlandsson, Nilsson, Tingkög, Västfjäll (2018) | d | a | b | b | 2 |
| Leonard (2018) | b | b | c | a | 3 |
| Foong, Hamid, Ibrahim, Haron (2018) | a | a | b | a | 4 |
| Ståhl, van Prooijen (2018) | d | b | c | a | 2 |
| Cavojová, Šrol, Jurkovic (2019) | b | a | c | a | 4 |
| Cavojová, Secara, Jurkovic, Šrol (2019) | b | b | c | a | 3 |
| Nilsson, Erlandsson, Västtjfäll (2019) | a | a | c | b | 4 |
| (1) Patel, Baker, Scherer (2019) | c | b | b | a | 2 |
| (2) Patel, Baker, Scherer (2019) | c | b | b | a | 2 |
| Betsch, Aßmann, Glöckner (2020) | b | a | c | c | 2 |
| Furnham, Grover (2020) | d | b | c | c | 0 |
| (1) Lowicki, Zajenkowski, van der Linden (2020) | b | b | b | a | 3 |
| (2) Lowicki, Zajenkowski, van der Linden (2020) | b | b | c | a | 3 |

^a^studies with longitudinal design, but data was extracted at the same observation time and studies were rated using the scale for cross-sectional studies

^b^case-control study, rated with the respective scale
