# Supplementary figures and images for: Meta-analyzing intelligence and religiosity associations: Evidence from the multiverse
Source: PLoS One. 2022 Feb 11;17(2):e0262699. doi: 10.1371/journal.pone.0262699 (PMC8836311; doi:10.1371/journal.pone.0262699)

**rstudent**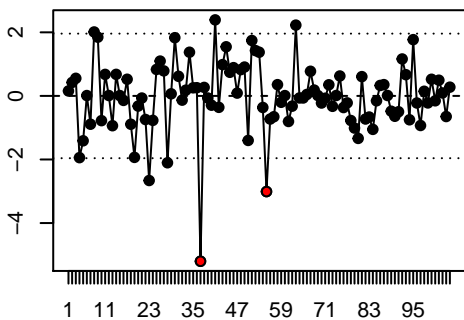**dffits**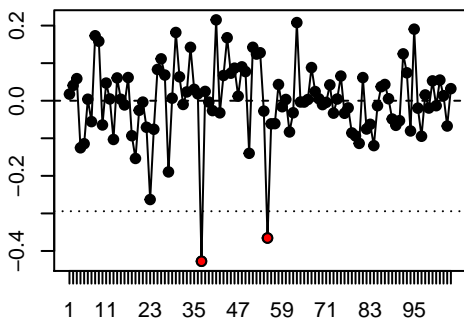**cook.d**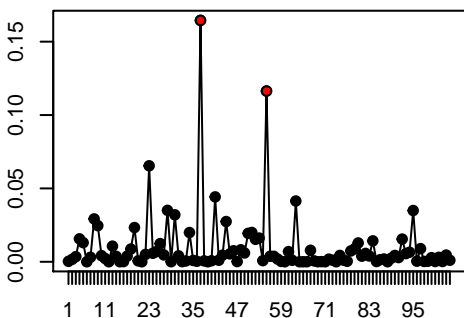**cov.r**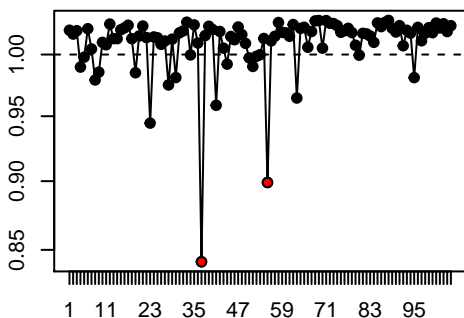**tau2.del**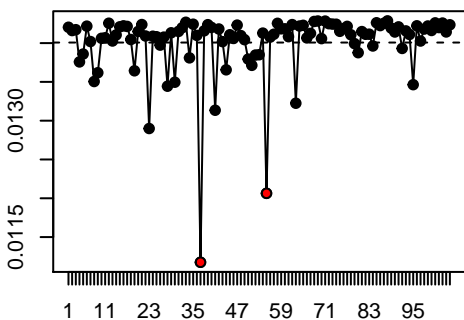**QE.del**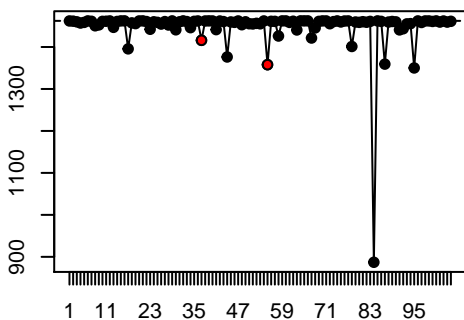**hat**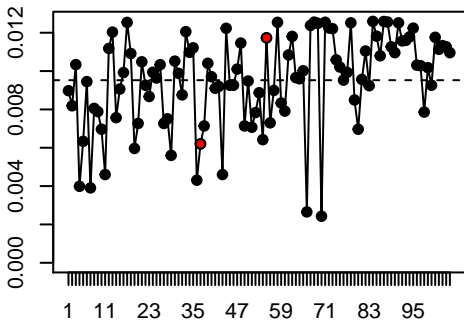**weight**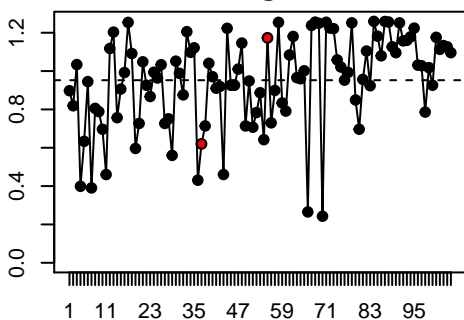

Supplement: S5 Appendix — (PDF) [file pone.0262699.s005.pdf]
