# Supplementary material for: Meta-analyzing intelligence and religiosity associations: Evidence from the multiverse
Source: PLoS One. 2022 Feb 11;17(2):e0262699. doi: 10.1371/journal.pone.0262699 (PMC8836311; doi:10.1371/journal.pone.0262699)
Supplement: S6 Appendix — (DOCX) [file pone.0262699.s006.docx]

**Table S6**

Zero-order and partial correlations among intelligence, religiosity, and education

|  | Intelligence and religiosity | | Intelligence and education | Education and religiosity | |
| --- | --- | --- | --- | --- | --- |
| Study | Zero-order correlations | Partial correlations^a^ | Zero-order correlations | Zero-order correlations | Partial correlations^b^ |
| Betsch, Aßman, Glöckner (2020) | -.22 | -.21 | .25 | -.08 | -.03 |
| Blanchard-Fields, Hertzog, Stein, & Pak (2001; Study 1) | .04 | .03 | .21 | .03 | .02 |
| Blanchard-Fields, Hertzog, Stein, & Pak (2001; Study 2) | -.33 | -.32 | .30 | -.08 | .02 |
| Drewelies, Deeg, Huisman, Gerstorf (2018; Study 1) | 0 | .02 | .19 | -.08 | -.08 |
| Drewelies, Deeg, Huisman, Gerstorf (2018; Study 2) | -.06 | .04 | .32 | -.08 | -.06 |
| Erlandsson, Nilsson, Tingkög, Västfjäll (2018) | -.23 | -.24 | .24 | .01 | .07 |
| Furnham, Grover (2020) | -.11 | -.11 | .04 | .02 | .02 |
| Ganzach & Gotlibovski (2013) | -.28 | -.27 | .57 | -.10 | .08 |
| Kanazawa (2010; Study 1) | -.12 | -.14 | .32 | .05 | .09 |
| Kanazawa (2010; Study 2) | -.14 | -.06 | .50 | -.17 | -.12 |
| Leonard (2018) | -.16 | -.15 | .08 | -.08 | -.07 |
| Lewis, Ritchie, & Bates (2011) | -.18 | -.11 | .41 | -.20 | -.14 |
| Pennycook, Cheyne, Seli, Koehler, & Fugelsang (2012; Study 1) | -.20 | -.19 | .22 | -.05 | -.01 |
| Pennycook, Cheyne, Seli, Koehler, & Fugelsang (2012; Study 2) | -.18 | -.16 | .27 | -.09 | -.04 |
| Pennycook, Cheyne, Barr, Koehler, & Fugelsang (2014a) | -.27 | -.27 | .23 | -.03 | .03 |
| Pollet & Schnell (2017) | -.22 | -.22 | -.01 | -.01 | -.01 |
| Ritchie, Gow, & Deary (2014) | -.12 | -.11 | .36 | -.05 | -.01 |
| Ross (2015) | -.19 | -.18 | .24 | -.05 | -.00 |
| Saribay & Yilmaz (2017) | -.10 | -.08 | .22 | -.12 | -.10 |
| Stahl & Prooijen (2018; Study 2) | -.17 | -.19 | .25 | .05 | .10 |
| Zuckerman & McPhetres (2016) | -.25 | -.25 | .28 | -.02 | .05 |

^a^ Partial correlations between intelligence and religiosity, controlling for education

^b^ Partial correlations between education and religiosity, controlling for intelligence
