# Supplementary material for: Meta-analyzing intelligence and religiosity associations: Evidence from the multiverse
Source: PLoS One. 2022 Feb 11;17(2):e0262699. doi: 10.1371/journal.pone.0262699 (PMC8836311; doi:10.1371/journal.pone.0262699)
Supplement: S7 Appendix — (DOCX) [file pone.0262699.s007.docx]

**Table S7**

Zero-order and partial correlations among intelligence, religiosity, and analytic style

|  | Correlations between intelligence and religiosity | | Correlations between intelligence and analytic style | Correlations between analytic style and religiosity | |
| --- | --- | --- | --- | --- | --- |
| Study | Zero-order correlations | Partial correlations^a^ | Zero-order correlations | Zero-order correlations | Partial correlations^b^ |
| Cavojová, Secară, Jurkovič, Šrol (2019) | -.12 | -.05 | .54 | -.15 | -.10 |
| Erlandsson, Nilsson, Tingkög, Västfjäll (2018) | -.23 | -.14 | .64 | -.2 | -.07 |
| Hartman et al. (2017; Study 3) | -.20 | -.14 | .36 | -.21 | -.15 |
| Leonard (2018) | -.16 | -.05 | .43 | -.28 | -.24 |
| Nilsson, Erlandsson, Västfjäll (2019) | -.16 | -.10 | .62 | -.13 | -.04 |
| Patel, Baker, Scherer (2019; Study 1) | -.08 | -.00 | .52 | -.15 | -.13 |
| Patel, Baker, Scherer (2019; Study 2) | -.13 | -.08 | .54 | -.12 | -.06 |
| Pennycook, Cheyne, Seli, Koehler, & Fugelsang (2012; Study 1) | -.19 | -.13 | .27 | -.26 | -.22 |
| Pennycook, Cheyne, Seli, Koehler, & Fugelsang (2012; Study 2) | -.17 | -.10 | .26 | -.30 | -.27 |
| Pennycook, Cheyne, Koehler, & Fugelsang (2013) | -.34 | -.24 | .30 | -.46 | -.40 |
| Pennycook, Cheyne, Barr, Koehler, & Fugelsang (2014a) | -.27 | -.18 | .45 | -.25 | -.15 |
| Pennycook, Cheyne, Barr, Koehler, & Fugelsang (2014b; Study 2) | -.23 | -.14 | .40 | -.27 | -.21 |
| Pennycook, Ross, Koehler, & Fugelsang (2016; Studies 1-4) | -.16 | -.02 | .54 | -.26 | -.21 |
| Razmyar & Reeve (2013) | -.27 | -.22 | .53 | -.17 | -.03 |
| Ross (2015) | -.19 | -.08 | .54 | -.22 | -.15 |
| Saribay & Yilmaz (2017) | -.10 | .01 | .42 | -.26 | -.24 |
| Shenav, Rand, & Greene (2012; Study 2) | -.06 | -.01 | .30 | -.18 | -.17 |
| Stahl & Prooijen (2018; Study 2) | -.17 | -.11 | .58 | -.14 | -.05 |
| Strimaitis (2018; Study 1) | -.04 | .11 | .56 | -.23 | -.18 |
| Strimaitis (2018; Study 2) | -.14 | -.06 | .42 | -.21 | -.16 |
| Zuckerman & McPhetres (2016) | -.25 | -.16 | .65 | -.20 | -.06 |

^a^ Partial correlations between intelligence and religiosity, controlling for education

^b^ Partial correlations between education and religiosity, controlling for intelligence
